# Cost-effectiveness of tarlatamab versus chemotherapy for patients with small-cell lung cancer after platinum-based chemotherapy in the United States and China

**DOI:** 10.3389/fphar.2026.1719846

**Published:** 2026-04-24

**Authors:** Xiaoju Liu, Qiuji Wu, Qiu Li, Yi Qin

**Affiliations:** 1 Division of Abdominal Tumor Multimodality Treatment, Cancer Center, West China Hospital, Sichuan University, Chengdu, China; 2 Department of Radiation Oncology, Sichuan Clinical Research Center for Cancer, Sichuan Cancer Hospital and Institute, Sichuan Cancer Center, Affiliated Cancer Hospital of University of Electronic Science and Technology of China, Chengdu, China

**Keywords:** cost-effectiveness, Markov model, price simulation, small-cell lung cancer, tarlatamab

## Abstract

**Background and Objective:**

Small-cell lung cancer (SCLC) is highly aggressive, and outcomes after relapse following platinum-based therapy remain poor. Tarlatamab, a DLL3-targeted bispecific T-cell engager, has demonstrated survival benefits in phase III trials. Given rising oncology expenditures, we evaluated the cost-effectiveness of tarlatamab versus chemotherapy from U.S. and Chinese payer perspectives.

**Methods:**

We developed a three-state Markov model using efficacy inputs reconstructed from DeLLphi-304. Parametric survival extrapolation and Bayesian model averaging were applied over a lifetime horizon with 1-month cycles. Direct medical costs included drug acquisition and administration, monitoring, management of grade ≥3 adverse events, subsequent therapies, supportive care, and end-of-life care; utilities were obtained from published sources. Costs were expressed in 2025 U.S. dollars. Base-case willingness-to-pay (WTP) thresholds were $150,000 per quality-adjusted life-years (QALYs) (U.S.) and three times *per capita* GDP (China). Price-simulation analyses evaluated tarlatamab price ranges in both settings and identified per-mg threshold prices that satisfied WTP criteria.

**Results:**

Tarlatamab yielded a 0.15-QALY gain versus chemotherapy. Incremental costs were $198,914.10 in the United States and $61,878.59 in China, corresponding to ICERs of $1,306,254.68 and $406,352.26 per QALY, respectively—both exceeding country-specific WTP thresholds. ICERs increased monotonically with the drug price. Deterministic and probabilistic sensitivity analyses indicated robustness; no plausible parameter variation reduced ICERs below prespecified thresholds. Subgroup results paralleled survival benefits, but all subgroup ICERs remained above WTP thresholds.

**Conclusion:**

At current prices, tarlatamab is not cost-effective for SCLC after platinum-based therapy in the United States or China. Achieving cost-effectiveness would require prices at or below the thresholds identified in the simulations. Affordability constraints are more stringent in China than in the United States. These findings inform value-based pricing, reimbursement negotiations, and equitable access strategies.

## Introduction

Lung cancer remains the leading cause of cancer mortality worldwide, with an estimated 2.5 million incident cases and 1.8 million deaths in 2022, underscoring its substantial global health burden (Bray et al., 2024). Among its subtypes, small-cell lung cancer (SCLC) accounts for approximately 15% of diagnoses and characterized by rapid proliferation and early metastatic spread (Megyesfalvi et al., 2023; Gazdar et al., 2017; George et al., 2015). Extensive-stage SCLC (ES-SCLC), which accounts for nearly two-thirds of cases, is particularly aggressive, and most patients present after the disease has extended beyond the hemithorax (Rudin et al., 2021). First-line regimens afford disease control for only 6–9 months; second-line therapy has changed little and continues to rely on cytotoxic agents with modest efficacy and poor tolerability (Megyesfalvi et al., 2023). Topotecan, historically the standard second-line option, achieves objective response rates of 7%–24% but is associated with substantial hematologic toxicity, limiting its use and adherence (O’Brien et al., 2006). Lurbinectedin, approved in the United States in 2020, prolongs progression-free survival (PFS) but has not demonstrated a significant overall survival (OS) benefit and still carries considerable hematologic toxicity (Subbiah et al., 2020).

Tarlatamab is the first bispecific T-cell engager (BiTE) approved for SCLC. It simultaneously engages delta-like ligand 3 (DLL3)—expressed in >80% of SCLC tumors and largely absent from normal tissues—and CD3 on T cells, thereby redirecting cytotoxic T lymphocytes to DLL3-positive tumor cells and inducing immune-mediated lysis (Giffin et al., 2021). This mechanism operates independently of checkpoint blockade and represents a novel immunotherapeutic strategy for DLL3-high tumors. Preliminary findings from the Phase II DeLLphi-301 trial, including an objective response rate of 40% and a median OS of 14.3 months in heavily pretreated patients, supported accelerated approval of tarlatamab by the U.S. Food and Drug Administration (FDA) in May 2024 (Ahn et al., 2023; FDA, 2025).

The pivotal, multicenter, randomized, open-label Phase III DeLLphi-304 trial enrolled 509 patients with platinum-refractory SCLC and confirmed these findings: median OS was 13.6 months with tarlatamab versus 8.3 months with chemotherapy (hazard ratio [HR], 0.60; 95% confidence interval [CI], 0.47–0.77; P < 0.001) (Mountzios et al., 2025). Median PFS was also longer with tarlatamab (4.2 vs. 3.7 months; HR, 0.71; 95% CI, 0.59–0.86; P = 0.002), accompanied by higher objective response rates (27% vs. 9%) and a longer duration of response. Safety was favorable: grade ≥3 adverse events (AEs) occurred in 54% of patients receiving tarlatamab versus 80% receiving chemotherapy, largely reflecting reduced hematologic toxicity. Cytokine release syndrome (CRS) occurred in approximately 50% of patients but was predominantly grade 1–2. Together with improvements in patient-reported symptoms such as dyspnea and cough, these results support tarlatamab as a potential new standard of care for second-line SCLC. In the United States (U.S.), the National Comprehensive Cancer Network (NCCN) Clinical Practice Guidelines for SCLC recommend tarlatamab for patients who have progressed after platinum-based chemotherapy (National Comprehensive Cancer Network, 2025). In China, the marketing application for tarlatamab has been accepted. The therapy was previously granted priority review for adults with ES-SCLC who had received at least two prior lines, including platinum-based chemotherapy (Center for drug evaluation, 2023).

Amid escalating oncology expenditures, evaluating the economic value of novel therapies is increasingly essential. Cost-effectiveness analysis compares incremental costs and health outcomes to estimate incremental cost-effectiveness ratios (ICERs), thereby informing reimbursement decisions, formulary inclusion, and broader health-policy development. In the United States, persistently high drug prices continue to limit affordability. In China, where >1.06 million new lung-cancer cases are diagnosed annually, the resulting financial burden places substantial pressure on the national health-insurance system (Zheng et al., 2022).

Using data from DeLLphi-304, this study evaluated the cost-effectiveness of tarlatamab versus chemotherapy as treatment for SCLC after platinum-based chemotherapy from both U.S. and Chinese payer perspectives. By clarifying key value drivers, the analysis aims to guide stakeholders toward equitable access and to ensure that the clinical benefits of tarlatamab translate into sustainable improvements across diverse healthcare ecosystems.

## Methods

This study followed the Consolidated Health Economic Evaluation Reporting Standards 2022 (Husereau et al., 2022; Supplementary Table S1) (Husereau et al., 2022).

### Model overview

We developed a three-state Markov model to assess the long-term economic impact of tarlatamab versus chemotherapy in SCLC after platinum-based therapy (Wan et al., 2019; Vijenthira et al., 2023). The model comprised three health states (Bray et al., 2024): PFS, with active treatment and ongoing monitoring (Megyesfalvi et al., 2023); progressed disease (PD), characterized by palliative and supportive care; and (Gazdar et al., 2017) death, an absorbing state (Supplementary Figure S1) (Williams et al., 2017). The analysis adopted the healthcare payer perspective in both the United States and China. Efficacy inputs were derived by digitizing Kaplan–Meier curves from the Phase III DeLLphi-304 trial and fitting parametric survival models for extrapolation (Supplementary Table S2) (Mountzios et al., 2025). The cycle length was 1 month, and the time horizon was lifetime (Ishak et al., 2013). Primary outcomes were quality-adjusted life-years (QALYs) and the ICER. Willingness-to-pay (WTP) thresholds were set at $150,000 per QALY in the United States, as recommended by Neumann et al. (2014), and at three times *per capita* gross domestic product (GDP,$40,247.01) in China (Yue et al., 2021; National Data). Markov model construction and statistical analyses were performed in R (version 4.4.1; http://www.r-project.org).

### Model structure

PFS and OS curves were extracted from DeLLphi-304 using WebPlotDigitize (Mountzios et al., 2025). Pseudo-individual patient data (pseudo-IPD) were reconstructed following (Hoyle and Henley, 2011). Reconstructed IPD were then fitted to standard and flexible parametric distributions (exponential, Weibull, Gompertz, log-logistic, log-normal, and generalized gamma). Model fit was evaluated using the Akaike and Bayesian information criteria and visual inspection. To account for structural uncertainty regarding the choice of extrapolation curve, AIC-weighted model averaging was employed rather than relying on a single parametric distribution (Supplementary Table S3; Supplementary Figure S2–S5) (Chu et al., 2023; Burnham and Anderson, 2002). Cycle-specific transition probabilities were derived directly from the fitted parametric survival functions, $S\left(t\right)$ ,in conjunction with background mortality data.

Transition probabilities were derived using a time-dependent approach consistent with the decomposition of partitioned survival data. The probability of remaining in the PFS state was calculated based on the conditional survival of the fitted PFS function, while the transition from PFS to death was set to the age- and sex-matched background mortality rate. This assumption was necessary to avoid double-counting cancer-related deaths in the absence of cause-specific individual patient data. Consequently, the transition from PFS to PD was computed as the residual probability of exiting the PFS state. To ensure the Markov trace accurately reproduced the fitted OS curves, the transition probability within the PD state was derived algebraically. Specifically, the probability of remaining in the progressed disease state ( $tp_{\text{PD}\to \text{PD}}$ ) was calculated at each cycle based on the net cohort flow required to match the target prevalence of the PD cohort ( $S_{\text{OS}}-S_{\text{PFS}}$ ). The transition probability from PD to death was then defined as the complement ( $1-tp_{\text{PD}\to \text{PD}}$ ). To ensure robustness, internal validity was strictly enforced by verifying that cycle-specific transition probabilities remained within logical bounds (0–1) and that the sum of state probabilities equaled 1(Supplementary Material). Furthermore, we validated the model’s accuracy by overlaying the Markov trace output against the fitted parametric survival curves, confirming reproduction of the input data with negligible error (Supplementary Figure S6).

### Costs and utility values

We included direct medical costs for ES-SCLC: drug acquisition and administration; routine monitoring and follow-up; management of grade ≥3 AEs with incidence ≥5% and CRS of all grades for the tarlatamab arm; subsequent systemic therapies after progression; best supportive care; and end-of-life care. Dosing assumptions used average body weight and body-surface-area values for U.S. and Chinese populations. U.S. unit drug costs were obtained from Centers for Medicare and Medicaid Services (CMS) Medicare Part B Average Sales Price files, and Chinese prices from the National Centralized Drug Procurement database (CMS.gov.Medicare, 2025; DrugDataexpy, 2024). Because tarlatamab is not yet marketed in China, the Chinese price was assumed at one-third of the U.S. price (Assistant Secretary for Planning and Evaluation, 2024; Lyu et al., 2025). To align clinical dosing protocols with our 1-month model cycle, we applied a time-weighted costing approach. The initial tarlatamab cycle captured the costs of three distinct administrations on days one, eight, and fifteen, alongside expenses for required intensive step-up monitoring. Subsequent maintenance cycles were normalized to two administrations per month with correspondingly reduced routine outpatient monitoring costs. Comparator chemotherapy regimens were similarly standardized to a 1-month equivalent to ensure synchronous cost accumulation.

Costs for AE management were sourced from the Healthcare Cost and Utilization Project, published pharmacoeconomic studies, and other public databases (Consumer Price Index, 2025; The Healthcare Cost and Utilization, 2025; Wu et al., 2021; National Data, 2025; Rui et al., 2022). For CRS specifically, we stratified resource utilization by severity based on the DeLLphi-304 trial protocols. Grade 1 and 2 events were modeled as outpatient management involving monitoring visits and supportive care, reflecting the clinical feasibility of shortened observation periods. Conversely, Grade 3 and higher events were assumed to require inpatient hospitalization and pharmacological intervention such as tocilizumab. Follow-up, supportive-care, and palliative-care costs were extracted from relevant literature and national healthcare databases (Consumer Price Index, 2025; National Bureau of Statistice, 2025; CMS.gov.Physician Fee Schedule, 2025; Shao et al., 2023; Medicare.gov, 2025; Criss et al., 2019; Gu et al., 2019; Zeng et al., 2012). To reflect real-world clinical practice and account for regional differences in drug accessibility, we modeled distinct post-progression treatment patterns for the US and China. While the proportion of patients receiving subsequent anti-tumor therapy was derived from the DeLLphi-304 trial, the specific regimens were tailored to align with local clinical practice guidelines. The remaining patients were assumed to receive Best Supportive Care (BSC), as detailed in Supplementary Table S4. To ensure temporal comparability, all costs were inflated to 2025 U.S. dollars using medical-specific indices; Chinese costs were additionally converted at the 2025 exchange rate ($1 = RMB 7.1371 (State Administration of Foreign Exchange, 2025).

Health state utility values representing health related quality of life were derived directly from a published real world observational study evaluating patients diagnosed specifically with small cell lung cancer, given the absence of quality-of-life data in the DeLLphi 304 trial. Baseline utilities were 0.70 for progression free survival, 0.60 for progressed disease, and 0 for death (Vedadi et al., 2021). Given the biological uniformity of extensive stage small cell lung cancer and its universal symptom burden, we assumed this disease specific utility weights were transferable across both the United States and Chinese modeled cohorts to address the current lack of robust country specific data. To rigorously address the uncertainty inherent in cross national utility transfer, we incorporated an exploratory sensitivity analysis by varying these baseline utility values by plus or minus 50%. Disutilities associated with severe adverse events presenting an incidence of 5% or greater were applied as one-time decrements in the first model cycle (Lee et al., 2015; Nafees et al., 2017; Yang et al., 2019). Furthermore, costs and utilities were discounted at an annual rate of 3% for the United States and 5% for China consistent with standard health economic guidelines (Yue et al., 2021; Su et al., 2021). Detailed parameters of costs and utilities are shown in Supplementary Tables S5, S6.

### Base case and price simulation

A base-case analysis was conducted to evaluate life-years (LYs), QALYs, total costs, and ICERs. For the United States, a price-simulation analysis examined a tarlatamab price range of $0–$2,000 per mg and estimated the drug prices that would achieve cost-effectiveness at WTP thresholds of $100,000 and $150,000 per QALY. Owing to the absence of market price data for tarlatamab in China, the baseline analysis assumed a reference price equal to one-third of the U.S. price and simulated a range of $0–$1567.49 per mg. This assumption reflects current Chinese policy, under which tarlatamab prices are expected to remain below U.S. market levels; accordingly, the upper bound was set to the U.S. market price and the lower bound to zero. In addition, the simulation estimated the cost-effective threshold price in China using a WTP of $95,907.44 per QALY, corresponding to three times the 2024 *per capita* GDP in Beijing, the highest-income region.

### Sensitivity analyses

Deterministic and probabilistic sensitivity analyses evaluated uncertainty in key parameters and structural assumptions. In the deterministic sensitivity analysis (DSA), parameters were varied one at a time within their reported 95% confidence intervals, or, when confidence intervals were unavailable, within ±20% of the base-case values. Additionally, an exploratory analysis was conducted with variations within ±50% of the base-case values. The results were summarized using tornado diagrams, which ranked the parameters based on their impact on the ICER. The unit price of tarlatamab was excluded from the DSA. For the probabilistic sensitivity analysis (PSA), gamma distributions were assigned to cost parameters, while beta distributions were used for probabilities and utility scores. In the PSA, 1,000 Monte Carlo simulations generated cost-effectiveness planes and acceptability curves to estimate the probability that tarlatamab is cost-effective across WTP thresholds.

### Subgroup analyses

Exploratory subgroup analyses were conducted for patients in the United States and China. In line with established practice and absent subgroup-specific Kaplan–Meier curves, the chemotherapy-arm survival was assumed identical across subgroups, and subgroup-specific hazard ratios from DeLLphi-304 were applied to adjust OS and PFS (You et al., 2024). ICERs were estimated for each subgroup using country-specific baseline tarlatamab prices and the cost-effective prices identified in the simulations.

### Scenario analysis

To rigorously evaluate model robustness, particularly regarding structural uncertainty that was assessed via deterministic scenarios rather than jointly within the PSA, we performed comprehensive scenario analyses. We replaced the base-case AIC-weighted model averaging with individual parametric survival distributions to assess the sensitivity of results to different extrapolation assumptions. Additionally, we interrogated the economic impact of our conservative base-case assumptions by incorporating costs for Grade 1–2 CRS and modeling adverse events as recurrent rather than one-time occurrences, while also testing the impact of shortening the time horizon to 5 and 10 years.

## Results

### Base case results

In the base-case analysis, compared with chemotherapy, tarlatamab extended life-years and yielded a gain of 0.15 QALYs. In the United States, the incremental cost was $198,914.10, resulting in an ICER of $1,306,254.68 per QALY gained. Under the base-case scenario for China, the cost of tarlatamab was assumed to be one-third of the U.S. price (Assistant Secretary for Planning and Evaluation, 2024), leading to an incremental cost of $61,878.59 and an ICER of $406,352.26 per QALY gained. Both ICERs exceeded the country-specific WTP thresholds (Table 1).

**TABLE 1:** Results of base-case analysis.

Parameter	US		China	
	Tarlatamab	Chemotherapy	Tarlatamab	Chemotherapy
Cumulative cost (USD)	248,766.23	49,852.12	72,393.49	10,514.90
Cumulative life years	1.54	1.39	1.54	1.39
Cumulative effect (QALY)	0.99	0.84	0.99	0.84
Incremental cost (USD)	198,914.10	​	61,878.59	​
Incremental effect(QALY)	0.15	​	0.15	​
ICER	1,306,254.68	​	406,352.26	​

ICER, incremental cost-effectiveness ratio; QALY, quality-adjusted life year; US, united states; USD, US, dollars.

### Price simulation

**FIGURE 1:**
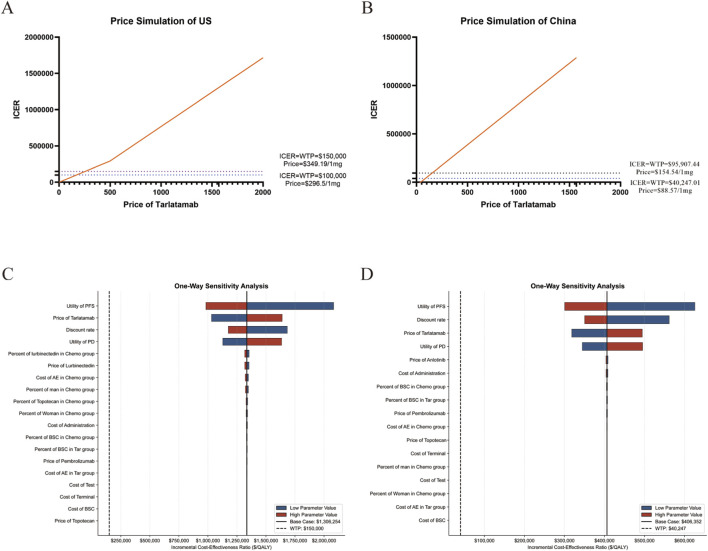
Results of price simulation and deterministic sensitivity analysis. **(A)** price simulation shows the ICER at various Tarlatamab prices in the United States. **(B)** Tornado diagram showing the results of a one-way sensitivity analysis in the United States. **(C)** Threshold analysis shows the ICER at various Tarlatamab prices in China. **(D)** Tornado diagram showing the results of a one-way sensitivity analysis in China. PD, progressive disease; PFS, progression-free survival; Chemo, Chemotherapy; Tar, Tarlatamab; AEs, adverse events; BSC, best supportive care.

ICER values rose monotonically with tarlatamab price. In the United States, within the $0–2,000/mg range, the ICER increased as price increased; at WTP thresholds of $100,000 and $150,000 per QALY, tarlatamab is cost-effective when priced below $296.50/mg and $349.19/mg, respectively (Figure 1A). In China, using a WTP threshold equal to three times the *per capita* GDP of Beijing ($95,907.44 per QALY), tarlatamab is cost-effective when priced below $154.54/mg; under a lower WTP of $40,247.01 per QALY, the cost-effective price is below $88.57/mg (Figure 1B).Collectively, these simulations indicate that targeted price adjustments could render tarlatamab cost-effective for patients with SCLC after platinum-based chemotherapy. We also conducted price simulations across different willingness-to-pay thresholds for both the United States and China (Supplementary Figure S7)

### Sensitivity analyses

**FIGURE 2:**
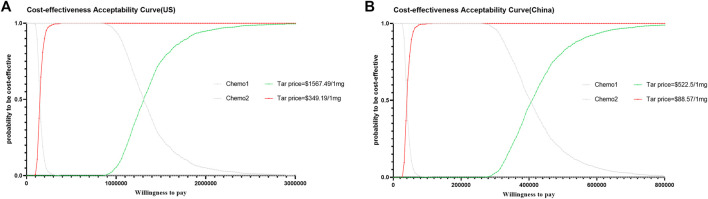
Probabilistic sensitivity analysis. **(A)** Cost-effectiveness acceptability curves in the United States. **(B)** Cost-effectiveness acceptability curves in China. Chemo, Chemotherapy; Tar, Tarlatamab.

Deterministic sensitivity analyses showed that ICER estimates were most sensitive to PFS utility, PD utility, and the discount rate. In the United States, the proportion and price of lurbinectedin use in the chemotherapy arm, as well as AEs costs, had moderate effects; other parameters had minor influence. In both countries, no parameter variation lowered the ICER below the prespecified WTP threshold (Figures 1C,D). Furthermore, we conducted an exploratory deterministic sensitivity analysis by varying the parameters by plus or minus 50 percent, as illustrated in Supplementary Figure S8. Probabilistic sensitivity analysis (1,000 Monte Carlo iterations) further demonstrated that the probability of cost-effectiveness increased with higher WTP thresholds but remained low at conventional thresholds, exceeding 50% only at substantially higher WTPs (Figures 2A,B). Given the dominant influence of the unit price of tarlatamab on ICER estimates, we generated cost-effectiveness acceptability curves and scatter plots for two simulated price scenarios in the United States and China (Supplementary Figure S6). Overall, results were robust to most inputs, but the economic viability of tarlatamab was highly price-dependent, with tighter affordability constraints in China.

### Subgroup analyses

Subgroup analyses assessed whether clinical characteristics affected economic outcomes. ICERs closely tracked survival benefits, with tarlatamab demonstrating more favorable cost-effectiveness in subgroups experiencing larger reductions in the risk of death. Relative to the overall population, lower ICERs were observed among women, White patients, and patients with brain metastases; nonetheless, all subgroup-specific ICERs exceeded WTP thresholds in both countries (Table 2). We therefore evaluated a price scenario that was cost-effective in the overall population; under this scenario, approximately half of subgroups exhibited a probability of cost-effectiveness greater than 50% (Supplementary Table S7). These findings underscore heterogeneity in the economic value of tarlatamab and may inform identification of patient groups most likely to achieve cost-effective benefit.

**TABLE 2:** Subgroup analysis.

Subgroups	HR for OS (95% CI)	HR for PFS (95% CI)	ICER1^a^ $/QALY (range)	CE Probability,%	ICER2^b^ $/QALY (range)	CE Probability,%
All patients	0.60 (0.47,0.77)	0.71 (0.59,0.86)	403,591.78 (207,552.63–662,011.41)	0%	124,797.95 (60,886.40–199,389.02)	0%
Age, years						
<65	0.57 (0.40,0.81)	0.77 (0.59,1.01)	360,109.83 (159,771.00–734,239.50)	0%	112,744.67 (46,223.70–221,430.36)	0%
≥65	0.67 (0.48,0.94)	0.65 (0.50,0.86)	405,445.37 (280,589.81–1,128,478.38)	0%	123,314.06 (88,966.63–331,253.62)	0%
Sex						
Male	0.70 (0.53,0.93)	0.77 (0.62,0.97)	470,348.09 (279,491.93–1043668.86)	0%	148,176.24 (90,542.82–318,871.33)	0%
Female	0.43 (0.26,0.72)	0.61 (0.42,0.87)	334,015.51 (196,614.17–691,012.38)	0%	99,916.98 (61,514.82–195,795.20)	0%
Region						
White	0.51 (0.37,0.70)	0.79 (0.61,1.02)	316,368.28 (204,232.05–550,270.04)	0%	99,155.14 (65,823.18–166,332.27)	0%
Asian	0.75 (0.50,1.11)	0.69 (0.51,0.94)	570,990.90 (279,628.03–2,295,185.81)	0%	176,792.77 (90,249.74–678,829.12)	0%
Prior anti-PD-(L)1 exposure						
Yes	0.61 (0.45,0.82)	0.67 (0.53,0.84)	428,457.93 (271,364.23–794,613.45)	0%	131,218.16 (85,569.87–235,064.47)	0%
No	0.65 (0.42,1.03)	0.89 (0.62,1.28)	381,285.18 (185,706.91-dominated^c^)	0%	122,812.07 (61,820.84-dominated)	0%
Chemotherapy-free interval						
<90 days	0.60 (0.43,0.84)	0.71 (0.54,0.94)	403,591.78 (240,023.31–831,237.59)	0%	124,797.95 (76,853.74–246,871.22)	0%
≥90 days	0.65 (0.45,0.93)	0.73 (0.56,0.95)	437,695.37 (246,191.71–1,071,213.12)	0%	136,301.18 (79,062.03–321,049.90)	0%
≥90 to <180 days	0.71 (0.46,1.10)	0.74 (0.52,1.04)	494,729.46 (233,166.91–2,208,017.26)	0%	154,842.90 (75,890.15–655,257.10)	0%
≥180 days	0.54 (0.29,1.03)	0.74 (0.49,1.12)	350,654.63 (168,451.09–1,556,151.05)	0%	108,893.44 (54,533.27–456,034.92)	0%
Brain metastases						
Yes	0.45 (0.31,0.65)	0.60 (0.45,0.80)	348,630.57 (225,881.22–588,560.17)	0%	104,084.93 (70,080.53–168,436.07)	0%
No	0.81 (0.58,1.13)	0.83 (0.64,1.08)	591,663.87 (283,177.16–3,173,519.26)	0%	189,851.85 (93,525.77–981,091.10)	0%
Liver metastases						
Yes	0.82 (0.57,1.18)	0.84 (0.62,1.14)	604,296.51 (266,061.58–5,546,933.80)	0%	194,410.65 (88,474.01–1,706,241.04)	0%
No	0.54 (0.39,0.75)	0.69 (0.54,0.88)	368,348.12 (236,911.35–662,676.73)	0%	113,040.18 (74,930.06–196,303.91)	0%
Chemotherapy						
Topotecan/Amrubicin	0.57 (0.44,0.75)	0.76 (0.62,0.94)	363,443.61 (244,086.02–611,741.04)	0%	113,537.92 (78,210.79–185,785.02)	0%
Lurbinectedin	0.81 (0.46,1.44)	0.56 (0.34,0.90)	754,776.00 (261,413.33-dominated)	0%	225,464.11 (83,348.62–225,464.11)	0%

Costs are in US, dollars.

CI, confidence interval; HR, hazard ratio; ICER, incremental cost-effectiveness ratio; OS, overall survival; PD-L1, programmed cell death ligand 1; QALY, quality-adjusted life year; US, united states; CE, cost effectiveness.

^a^ ICER1 is calculated based in the US; price of Tarlatamab = $ 1567.49/1 mg; WTP = $150,000.00.

^b^ ICER2 is calculated based in the China; price of Tarlatamab = $ 522.50/1mg; WTP = $40247.01.

^c^ “Dominated” reveals that a plan is an absolute disadvantaged one.

### Scenario analysis

The scenario analysis revealed significant outcome variability based on modeling assumptions. The log normal distribution provided the most optimistic incremental cost effectiveness ratios of 172,409 dollars and 52,137 dollars per quality adjusted life year for the United States and China respectively, whereas the Gompertz distribution yielded considerably higher respective ratios of 712,384 dollars and 222,080 dollars. Applying a standardized mortality ratio of 1.5 to the progression free survival state to address potential pre-progression mortality resulted in minimal baseline fluctuations, confirming the robustness of the base case conclusions. Regarding safety parameters, incorporating recurrent adverse events increased the respective ratios to approximately 1.29 million dollars and 402,307 dollars. Including grade one and two cytokine release syndrome produced the least favorable safety profiles with ratios rising to 1.34 million dollars and 435,227 dollars respectively. Furthermore, restricting the time horizon to five or 10 years drastically elevated the cost effectiveness ratios across both cohorts as detailed in Supplementary Table S8.

## Discussion

SCLC accounts for approximately 15% of lung cancers and is characterized by markedly aggressive biology, early, widespread metastasis, and a high risk of recurrence. For patients who relapse after platinum-based therapy, treatment options remain limited and prognosis is poor. Tarlatamab, a DLL3-targeted BiTE, is a novel therapeutic strategy for relapsed or refractory SCLC. Clinical trials have shown that tarlatamab can significantly extend OS and improve quality of life in some patients, underscoring its potential as a breakthrough therapy. These benefits are notable given the limited efficacy of conventional chemotherapy. Nevertheless, the high cost of emerging immunotherapies and targeted agents remains a major barrier to broad adoption (Rivera-Concepcion et al., 2022). Accordingly, evaluating the cost-effectiveness of tarlatamab across distinct healthcare systems, such as those of the United States and China, is essential. This cross-national analysis provides evidence to inform value-based pricing and reimbursement decisions.

Our findings indicate that tarlatamab confers clinically meaningful benefits compared with chemotherapy. Median OS was 13.6 months with tarlatamab versus 8.3 months with chemotherapy (increment, 5.3 months). QALYs were 0.99 with tarlatamab and 0.84 with chemotherapy, reflecting gains in both survival and health-related quality of life. Despite these clinical advantages, our cost-effectiveness analysis showed that the current price of tarlatamab substantially limits its economic viability. Our subgroup analysis revealed that the cost-effectiveness profile of tarlatamab is highly consistent across diverse patient populations, including stratifications by age, sex, and metastatic sites. Although numerical variations in ICERs were observed—reflecting the inherent prognostic heterogeneity of SCLC—the ICERs in all analyzed subgroups remained significantly above the willingness-to-pay thresholds in both the US and China. They indicate that patient stratification alone is insufficient to mitigate the economic burden of tarlatamab. Unlike some therapies where restricting use to a “high-responder” subgroup might achieve cost-effectiveness, the acquisition cost of tarlatamab is the dominant driver of the ICER, overshadowing differences in clinical benefit across subgroups. Consequently, payers and policymakers should prioritize value-based pricing negotiations to achieve broad affordability, rather than attempting to limit coverage to specific patient subsets.

In the United States, the incremental cost of tarlatamab versus chemotherapy was $198,914.10, with the ICER exceeding the country-specific WTP threshold; price-simulation analyses indicated that tarlatamab would be considered cost-effective only if its price were reduced by approximately 80%. The assumption that the Chinese acquisition cost of tarlatamab is one-third of the US price was supported by an analysis of comparable immuno-oncology agents. Currently, imported Immune checkpoint inhibitors such as atezolizumab and pembrolizumab are priced in China at approximately 39%–42% of their US levels (DrugDataexpy, 2024; ASP Pricing Files, 2024). Our 33% assumption reflects a conservative estimate consistent with this range and potential National Reimbursement Drug List (NRDL)-driven discounts. In China, the incremental cost was $61,878.59, with the ICER also above the threshold; achieving cost-effectiveness would require a national price reduction of about 95% and approximately 90% in Beijing. Collectively, these simulations indicate that targeted price adjustments could render tarlatamab cost-effective for patients with SCLC after platinum-based chemotherapy.

In recent years, numerous studies have evaluated the cost-effectiveness of immunotherapies for ES-SCLC, generating a substantial evidence base. Immunotherapy–chemotherapy combinations, particularly those involving PD-1/PD-L1 inhibitors, have demonstrated survival benefits in clinical trials; however, achieving cost-effectiveness remains a consistent challenge across different economic regions. In IMpower133, atezolizumab plus chemotherapy improved survival; however, subsequent cost-effectiveness analyses in both the United States and China found ICERs exceeding their respective willingness-to-pay thresholds (Zhou et al., 2019; Li et al., 2019). Similarly, CASPIAN demonstrated the clinical benefit of durvalumab plus chemotherapy, but economic assessments placed the ICER near the threshold in high-income countries and prohibitively high in low- and middle-income countries (Ding et al., 2021; Tong et al., 2022). Although direct comparative cost-effectiveness studies of tarlatamab are not yet available, current evidence suggests that its economic challenges—driven primarily by high pricing—mirror those observed for PD-1/PD-L1 inhibitors. These findings highlight that although novel immunotherapies improve prognosis, translation from clinical benefit to economic value remains contingent on price negotiation, reimbursement policy, and innovative payment models (Levaggi et al., 2014; Yi et al., 2025).

In our base-case United States analysis, the ICER of tarlatamab at its current list price substantially exceeded the assumed WTP threshold. Given the Inflation Reduction Act, which expands Medicare’s authority to negotiate drug prices, future reductions in tarlatamab’s price—via value-based payment models or direct negotiation—could render it cost-effective (Inflation Reduction Act and Medicare, 2024). In China, however, the affordability challenge is even greater: a price reduction of approximately 95% would be required to achieve cost-effectiveness. Notably, several immune checkpoint inhibitors have obtained 40%–70% price reductions through NRDL negotiations, markedly improving economic viability (Yi et al., 2025). If tarlatamab were incorporated into reimbursement via similar strategies—price negotiation, tiered reimbursement, and risk-sharing mechanisms—its economic feasibility could improve. Substantial regional disparities in economic development persist; in wealthier regions (e.g., Beijing and Shanghai), higher *per capita* GDP places tarlatamab closer to the cost-effectiveness threshold, whereas adoption remains more restricted in low- and middle-income regions.

This study has several strengths. First, to our knowledge, this is the first evaluation of the cost-effectiveness of tarlatamab versus chemotherapy in patients with SCLC progressing after platinum-based therapy, conducted in parallel for the United States and China. These findings provide new evidence to guide cross-national drug pricing and policy. Second, we implemented a rigorously specified Markov model and assessed robustness through extensive sensitivity and scenario analyses. Furthermore, we incorporated price-simulation analyses, offering quantitative inputs for reimbursement negotiation and pricing in a rapidly evolving immunotherapy landscape.

This study also has limitations. First, because long-term follow-up data were unavailable, survival beyond the observation period was extrapolated using parametric functions. To mitigate uncertainty, we compared multiple models and selected the distribution with the best visual fit. We acknowledge that the inability to formally test the proportional hazards assumption constitutes a methodological limitation necessitated by our reliance on published summary hazard ratios rather than individual patient data. Second, most cost inputs and healthcare resource-use parameters were drawn from published literature or other cancer types and may not fully reflect real-world practice. Nevertheless, after inflation adjustment and sensitivity analyses, findings remained robust. Third, because trial-based health-related quality-of-life data were unavailable, we used external sources, which may limit generalizability. However, varying utility values did not alter the conclusion regarding whether the ICER exceeded the WTP threshold. Finally, owing to limited data on subsequent therapies, we made assumptions about post-progression treatment based on trial reports and guidelines, which may differ from actual practice.

## Conclusion

For patients with SCLC who relapse after platinum-based therapy, tarlatamab offers substantial survival benefits; however, at the current price it is unlikely to be cost-effective in either the United States or China. Price reductions of approximately 80% in the United States and 95% in China would be required to meet country-specific WTP thresholds. Given its clear clinical potential, appropriate price adjustments and supportive reimbursement policies are essential to improve affordability and access. These results provide evidence to support clinical decision-making and inform policy on pricing and allocation of limited healthcare resources.

## Data Availability

The original contributions presented in the study are included in the article/Supplementary Material, further inquiries can be directed to the corresponding author.
