# Supplementary material for: Cost-effectiveness of tarlatamab versus chemotherapy for patients with small-cell lung cancer after platinum-based chemotherapy in the United States and China
Source: Front Pharmacol. 2026 Apr 24;17:1719846. doi: 10.3389/fphar.2026.1719846 (PMC13153070; doi:10.3389/fphar.2026.1719846)
Supplement: Supplementary file 1 [file Supplementaryfile1.docx]

# **Supplementary Methods**

## **Overview and Transitions from the Progression-Free Survival (PFS) State**

The Markov model was constructed using a time-dependent approach designed to align strictly with the partitioned survival analysis (PartSA) framework. The model inputs were the fitted parametric survival functions for progression-free survival, $S_{PFS}\left( t \right)$, and overall survival, $S_{OS}\left( t \right)$, derived from the reconstructed individual patient data (IPD) of the DeLLphi-304 trial. For a given cycle length $u$(1 month), the transition probabilities were derived algebraically at each cycle $t$. For the PFS state, the probability of remaining in the state, $tp_{PFS\to PFS}\left( t \right)$, was calculated as the conditional survival probability $S_{PFS}\left( t+u \right)/S_{PFS}\left( t \right)$. To avoid potential double-counting of cancer-related mortality in the absence of cause-specific event data, the transition from PFS to death, $tp_{PFS\to Death}\left( t \right)$, was constrained to the age- and sex-matched background mortality rate ($r_{bg}$). Consequently, the transition to progressed disease (PD), $tp_{PFS\to PD}\left( t \right)$, was computed as the residual probability: $1-tp_{PFS\to PFS}\left( t \right)-tp_{PFS\to Death}\left( t \right)$.

## **Derivation of Transitions within the Progressed Disease (PD) State**

To ensure the Markov cohort trace accurately reproduced the fitted overall survival curves without requiring numerical calibration, the transition probabilities for the PD state were derived based on cohort flow dynamics. Specifically, we calculated the probability of a patient remaining in the PD state, $tp_{PD\to PD}\left( t \right)$, by balancing the inflow of newly progressing patients against the target prevalence defined by the survival functions. The number of patients residing in the PD state at any time $t$ is defined as$N_{PD}\left( t \right)=S_{OS}\left( t \right)-S_{PFS}\left( t \right)$. The inflow of new patients entering PD from the PFS state during the cycle is$N_{Inflow}\left( t \right)=S_{PFS}\left( t \right)\times tp_{PFS\to PD}\left( t \right)$.

The target population for the PD state at the subsequent cycle ($t+u$) comprises patients who remained in PD plus the new inflow. Therefore, the number of patients who must "stay" in PD is derived as the target population at $(t+u)$minus the inflow. The transition probability is expressed as:

$$tp_{PD\to PD}\left( t \right)=\frac{\left[ S_{OS}\left( t+u \right)-S_{PFS}\left( t+u \right) \right]-\left[ S_{PFS}\left( t \right)\times tp_{PFS\to PD}\left( t \right) \right]}{S_{OS}\left( t \right)-S_{PFS}\left( t \right)}$$

The transition probability from PD to death was then defined as the complement: $tp_{PD\to Death}\left( t \right)=1-tp_{PD\to PD}\left( t \right)$

## **Internal Validity and Consistency Checks**

To verify the structural integrity of the model and the accuracy of the derived probabilities, rigorous internal consistency checks were implemented at every simulation cycle. First, we verified that all derived transition probabilities remained within the logical bounds of [0, 1]. Second, a summation checks confirmed that the sum of state occupancies (PFS + PD + Death) equaled 1.0 at all-time points. Finally, a trace validation was performed by overlaying the aggregated Markov model output (sum of PFS and PD states) against the input fitted parametric survival curves (BMA model).

## **Cost**

To ensure temporal comparability and rigorous traceability, all economic inputs were standardized to 2025 United States dollars using a systematic 'inflate-then-convert' valuation method. For cost inputs derived from Chinese historical literature or databases, we applied a two-step conversion process where historical Renminbi costs were first inflated to the 2025 base year using the annual medical-specific Health Care Consumer Price Index published by the National Bureau of Statistics of China. These inflation-adjusted values were subsequently converted to United States dollars using the projected 2025 exchange rate of 7.1371 Renminbi per dollar. An exception was made for the pricing of tarlatamab in China, which was derived directly as one-third of the United States price without additional inflation adjustment, given that the drug is not yet marketed in the region. United States costs were similarly adjusted to the 2025 base year using the Medical Care component of the Consumer Price Index from the United States Bureau of Labor Statistics where necessary. Furthermore, we stratified our cost sources by category to reflect the payer perspective accurately: drug acquisition costs were derived from the National Centralized Drug Procurement database and Centers for Medicare and Medicaid Services files to represent actual reimbursement prices ; routine monitoring and medical service fees were based on government-regulated provincial pricing schedules and physician fee schedules ; and costs for managing adverse events and providing best supportive care were extracted from recent peer-reviewed pharmacoeconomic literature. The specific original price year for each component is documented in the supplementary materials.

## **Post-Progression Treatment Pathways and Assumptions**

Upon disease progression, patients transitioning to subsequent care pathways were modeled based on precise utilization rates from the DeLLphi-304 trial. Specifically, 44% of patients in the tarlatamab group and 49% in the chemotherapy group received active subsequent anticancer therapy. To reflect clinical realities regarding prior treatment exposure, a 29% utilization rate for pembrolizumab was incorporated universally across these active therapy cohorts to precisely account for the proportion of trial patients who were naïve to prior immune checkpoint inhibitors. The clinical allocation logic for post-progression care was maintained consistently across both the United States and China models to ensure structural integrity. Patients who did not receive active subsequent systemic therapy were exclusively allocated to Best Supportive Care. Furthermore, End-of-Life care costs were applied uniformly as a one-time terminal cost to all patients upon transitioning to the death state. For the cohort receiving active subsequent anti-tumor therapy, we did not apply a fixed treatment duration constraint. Instead, the per-cycle costs of specific subsequent therapies were applied continuously for the duration patients remained in the progressed disease state, as governed by the Markov model transition probabilities. This dynamic approach reflects the clinical reality of maintaining treatment until subsequent progression or unacceptable toxicity. The exact country-specific regimen compositions and their explicit intra-cohort percentage weights are detailed in Supplementary Table S4.

## **Subgroup Survival Derivation and Probabilistic Sensitivity Analysis**

For the subgroup analyses, the survival function for the tarlatamab arm was calculated by raising the baseline survival function to the power of the respective hazard ratio, expressed mathematically as

$$S_{subgroup}\left( t \right)=S_{baseline}\left( t \right)^{HR}$$

During the ten thousand Monte Carlo iterations executed for the probabilistic sensitivity analyses, the subgroup specific hazard ratios were strictly maintained as fixed deterministic values to scale the baseline survival probabilities. Concurrently, all other input parameters including direct medical costs and health state utilities were varied probabilistically following their established base case distributions such as gamma distributions for costs and beta distributions for utilities. The cost effectiveness within each subgroup was subsequently evaluated against the specific willingness to pay thresholds of 150,000 United States dollars per quality adjusted life year for the United States cohort and 95,907.44 United States dollars for the China cohort.

## **Scenario Analysis**

In the scenario analysis addressing cytokine release syndrome (CRS), model inputs were explicitly stratified by severity and timing. Costing assumptions reflected the DeLLphi-304 trial protocols: Grade 1 and 2 events were modeled as outpatient management (monitoring visits, labs, and supportive care) given the feasibility of reduced monitoring duration (6–8 hours), whereas Grade 3 and 4 events reflected inpatient management intensity (including ICU admission and tocilizumab). Regarding health outcomes, disutility weights were converted to QALY losses assuming a duration of 2 days (the median time to resolution in the trial). All CRS-related costs and disutilities were applied as a one-time decrement in the first model cycle.

In the scenario analysis evaluating the impact of adverse events, disutility application was stratified by treatment arm to reflect distinct toxicity profiles. Adverse events such as anemia, neutropenia, and thrombocytopenia were modeled as recurrent events. Consequently, the associated disutilities were applied in each model cycle throughout the active treatment phase. The duration of this phase was parameterized based on the DeLLphi-304 trial results, which indicated a median treatment duration of 2.5 months (range: 0.0 to 14.7 months) for patients receiving topotecan, lurbinectedin, or amrubicin. Based on this median duration, the model applied recurrent disutilities for approximately 4 cycles in the base case analysis. In contrast, cytokine release syndrome was modeled as a one-time event applied in the first cycle only. This specific implementation aligns with clinical data indicating that 99% of cytokine release syndrome events occurred during the initial step-up dosing period and resolved rapidly without recurrence.
