# Supplementary material for: Cost-effectiveness of tarlatamab versus chemotherapy for patients with small-cell lung cancer after platinum-based chemotherapy in the United States and China
Source: Front Pharmacol. 2026 Apr 24;17:1719846. doi: 10.3389/fphar.2026.1719846 (PMC13153070; doi:10.3389/fphar.2026.1719846)
Supplement: Supplementary file 2 [file Supplementaryfile2.docx]

# **Cost-Effectiveness of Tarlatamab Versus Chemotherapy for Patients with Small-Cell Lung Cancer After Platinum-Based Chemotherapy in the United States and China**

**Figure S1 Model Structure**

**Figure S2 Survival plots for goodness-of-fit of parametric survival models for K-M curve of OS in patients with Tarlatamab.**

**Figure S3 Survival plots for goodness-of-fit of parametric survival models for K-M curve of OS in patients with Chemotherapy**

**Figure S4 Survival plots for goodness-of-fit of parametric survival models for K-M curve of PFS in patients with Tarlatamab**

**Figure S5 Survival plots for goodness-of-fit of parametric survival models for K-M curve of PFS in patients with Chemotherapy**

**Figure S6 Deterministic sensitivity analysis**

**Figure S7 Scatter plots for probabilistic sensitivity analysis**

**Table S1 CHEERS 2022 Checklist**

**Table S2 Baseline characteristics of patients in DeLLphi-304**

**Table S3 Model choice for all survival data**

**Table S4 Basic parameters input to the model (US)**

**Table S5 Basic parameters input to the model (China)**

**Table S6 Subgroups**

**Table S7 Scenario Analysis**

**Table S8 Scenario Analysis**

# **Figure S1 Model Structure**


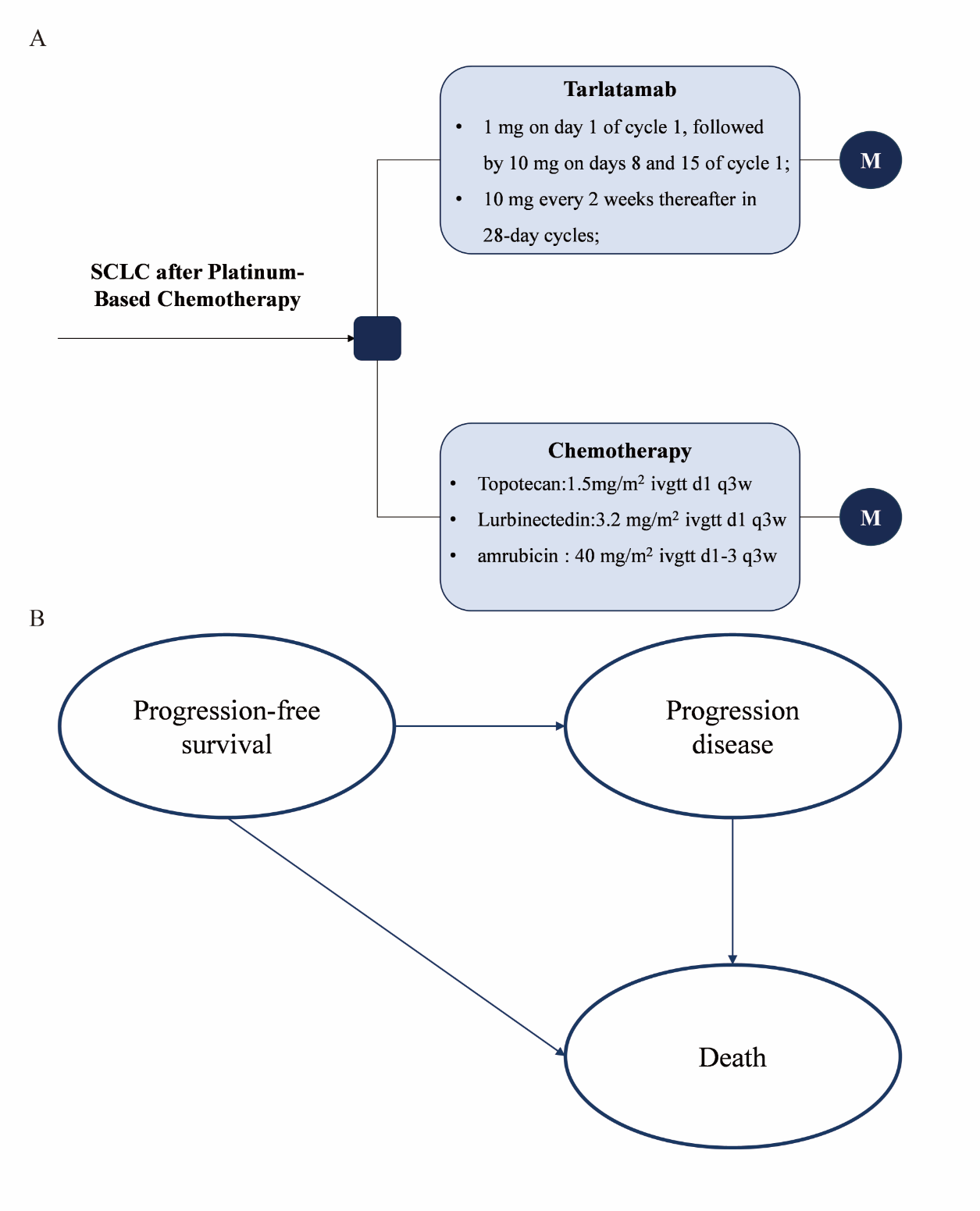


SCLC, small-cell lung cancer

# **Figure S2 Survival plots for goodness-of-fit of parametric survival models for K-M curve of OS in patients with Tarlatamab**


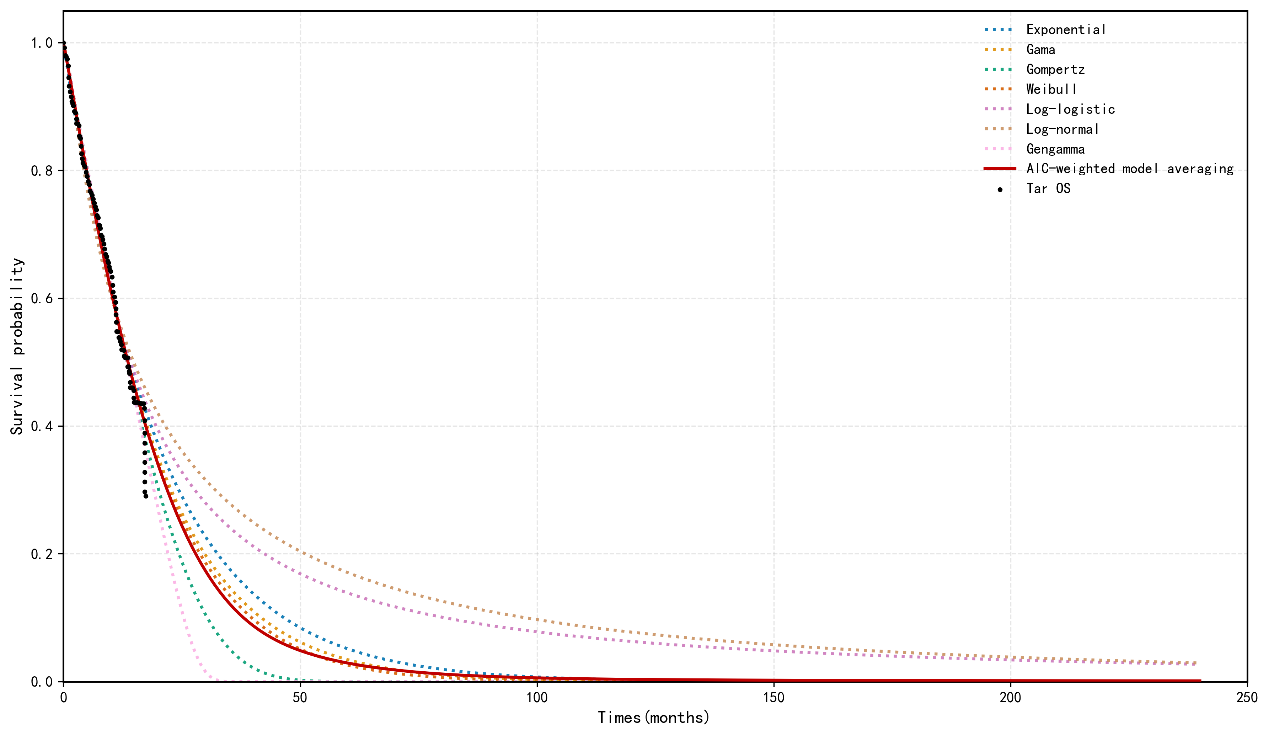


Tar, Tarlatamab; OS, overall survival, BMA, Bayesian model averaging

# **Figure S3 Survival plots for goodness-of-fit of parametric survival models for K-M curve of OS in patients with Chemotherapy**


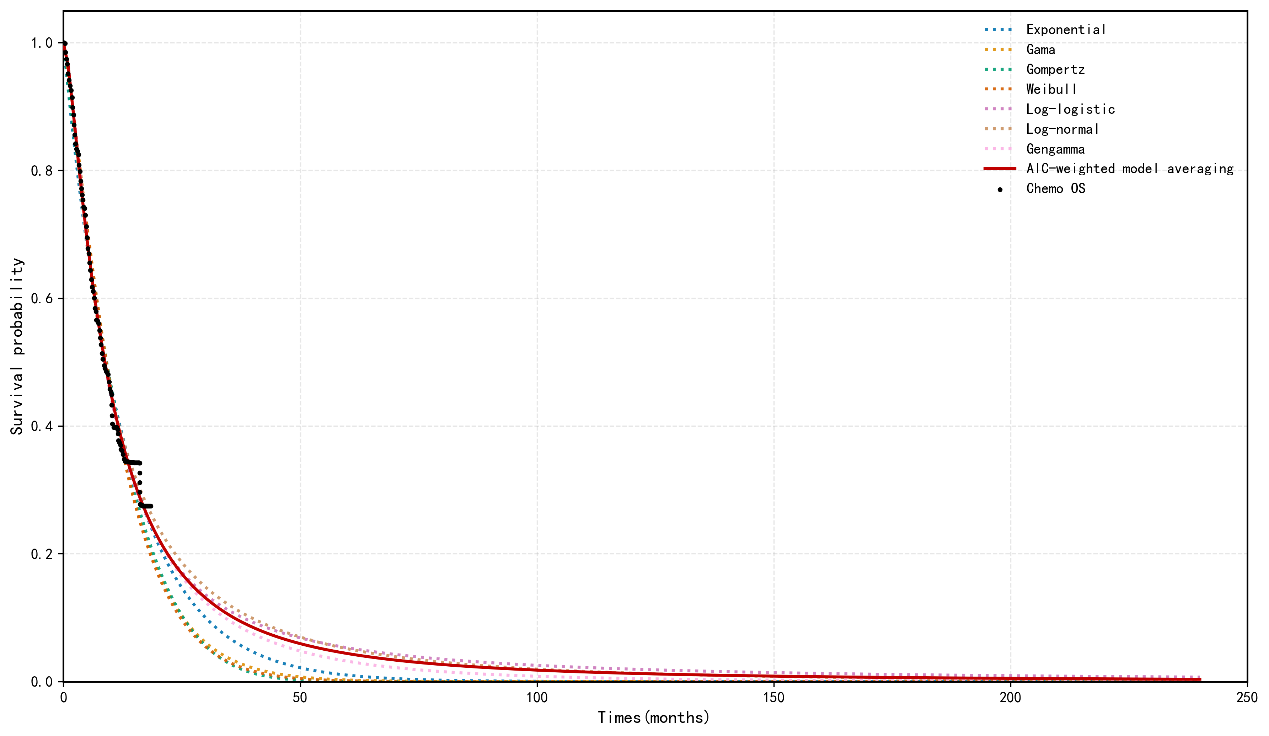


Chemo, Chemotherapy; OS, overall survival; BMA, Bayesian model averaging

# **Figure S4 Survival plots for goodness-of-fit of parametric survival models for K-M curve of PFS in patients with Tarlatamab**


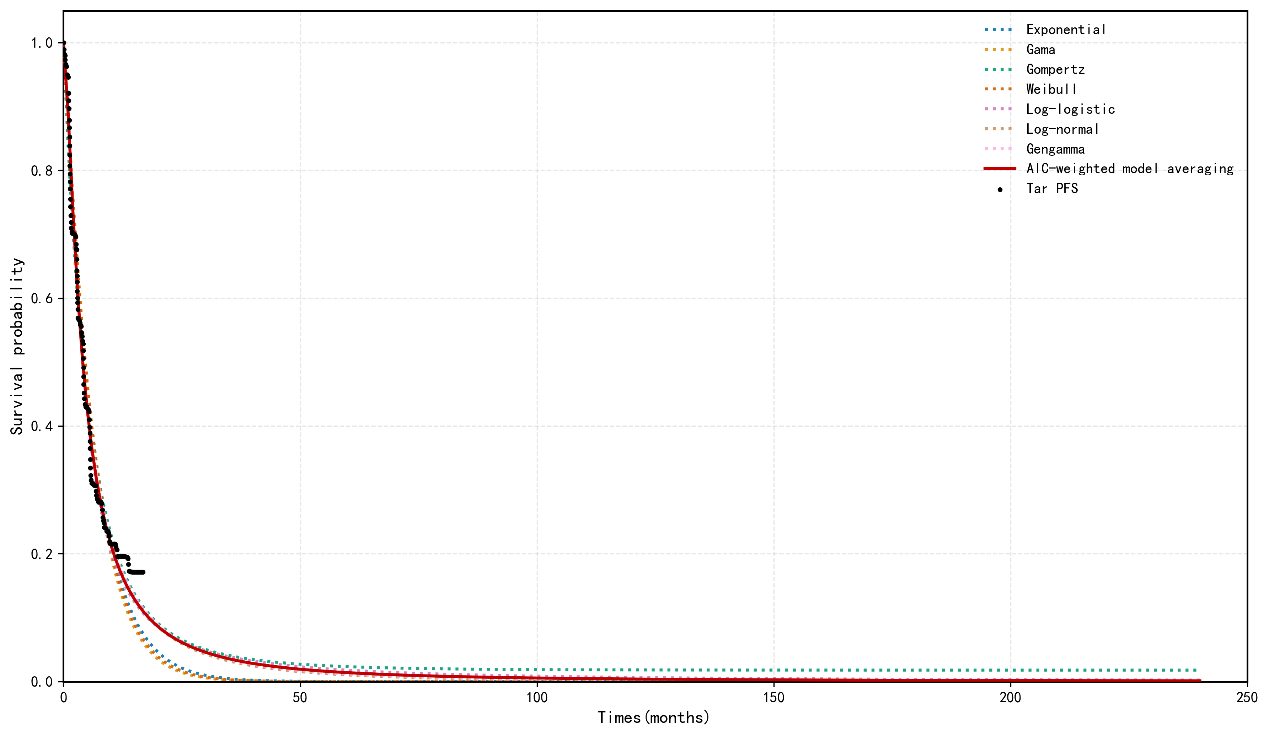


Tar, Tarlatamab; PFS progression-free survival; BMA, Bayesian model averaging

# **Figure S5 Survival plots for goodness-of-fit of parametric survival models for K-M curve of PFS in patients with Chemotherapy**

**
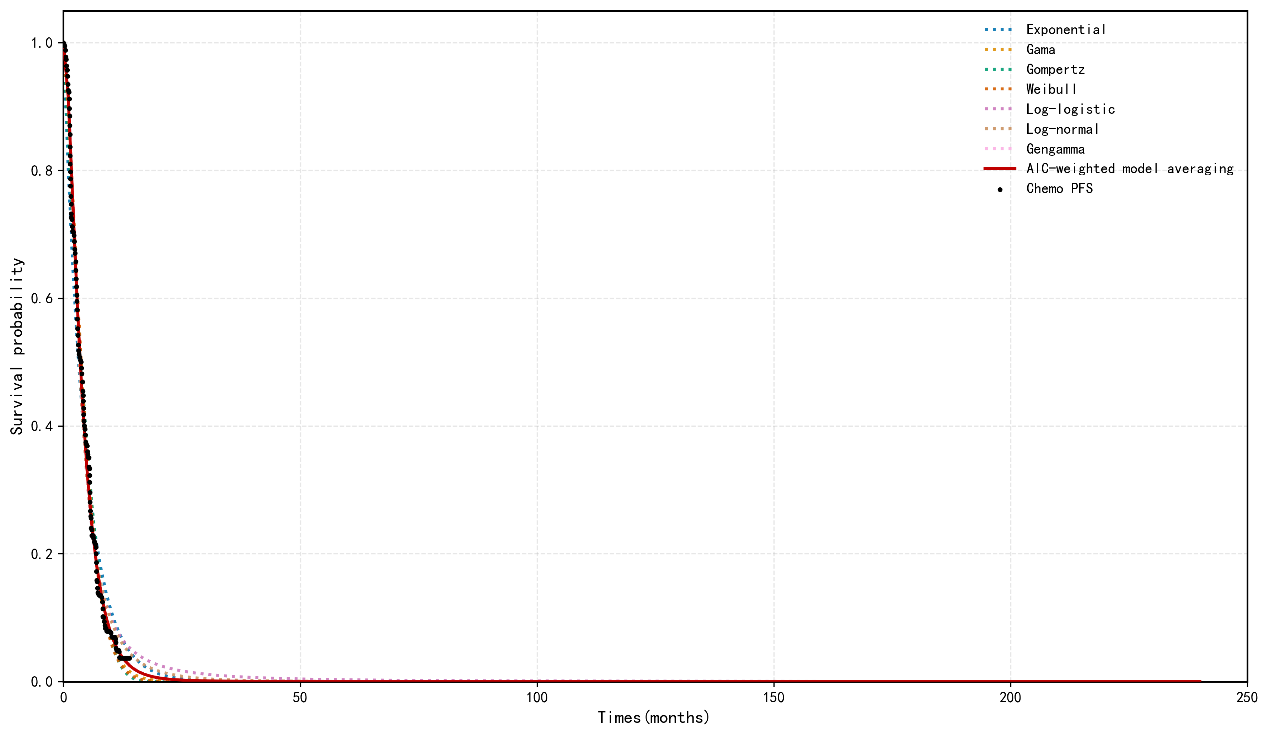
**

Chemo, Chemotherapy; PFS, progression-free survival; BMA, Bayesian model averaging

# **Figure S6 Validation of Internal Consistency**


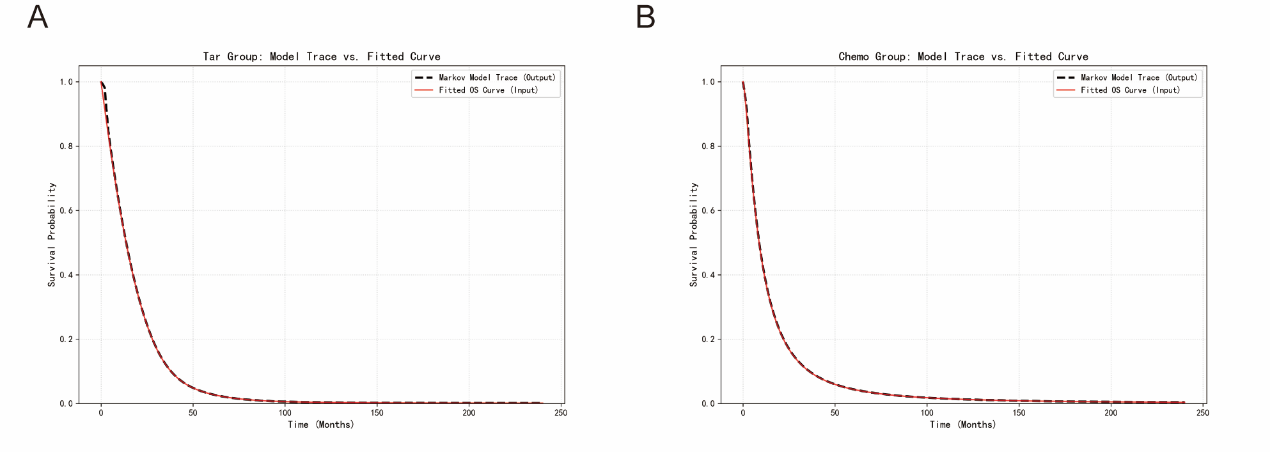
 Comparison of the Markov model trace (dashed line) versus the fitted parametric survival curve (solid line). (A) Tarlatamab group;(B) Chemotherapy group.

# **Figure S7 Scatter plots for probabilistic sensitivity analysis**


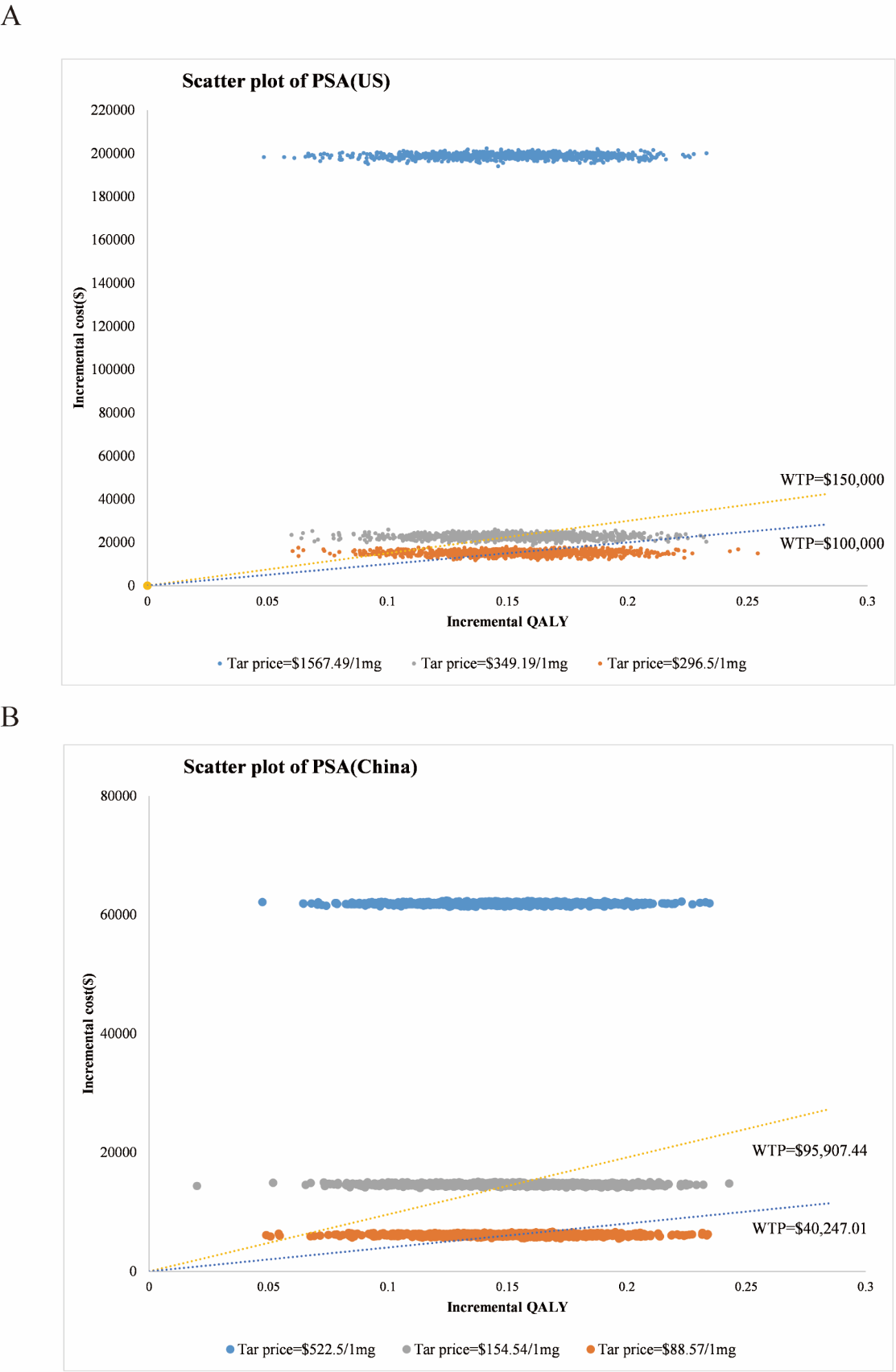


(A) Scatter plots for probabilistic sensitivity analysis in US (B) Scatter plots for probabilistic sensitivity analysis in China;

# **Figure S8 Deterministic sensitivity analysis**


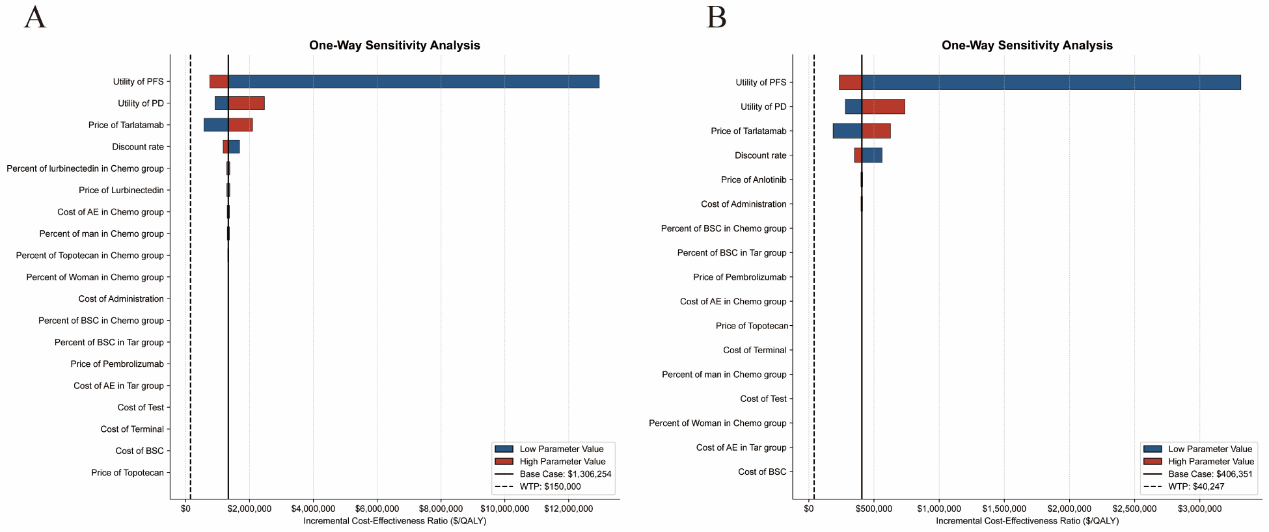


Results of deterministic sensitivity analysis (A) Tornado diagram showing the results of a one-way sensitivity analysis in the United States (±50%)(B) Tornado diagram showing the results of a one-way sensitivity analysis in China(±50%). PD, progressive disease; PFS, progression-free survival; Chemo, Chemotherapy; Tar, Tarlatamab ;AEs, adverse events; BSC, best supportive care.

# **Table S1 CHEERS 2022 Checklist**

|  | **Item** | **Guidance for Reporting** | **Reported in section** |
| --- | --- | --- | --- |
| **TITLE** | | |  |
| Title | 1 | Identify the study as an economic evaluation and specify the interventions being compared. | √ |
| **ABSTRACT** | | |  |
| Abstract | 2 | Provide a structured summary that highlights context, key methods, results and alternative analyses. | √ |
| **INTRODUCTION** | | |  |
| Background and objectives | 3 | Give the context for the study, the study question and its practical relevance for decision making in policy or practice. | √ |
| **METHODS** | | |  |
| Health economic  analysis plan | 4 | Indicate whether a health economic analysis plan was developed and  where available. |  |
| Study population | 5 | Describe characteristics of the study population (such as age range, demographics, socioeconomic, or clinical characteristics). | √ |
| Setting and location | 6 | Provide relevant contextual information that may influence findings. |  |
| Comparators | 7 | Describe the interventions or strategies being compared and why chosen. | √ |
| Perspective | 8 | State the perspective(s) adopted by the study and why chosen. | √ |
| Time horizon | 9 | State the time horizon for the study and why appropriate. | √ |
| Discount rate | 10 | Report the discount rate(s) and reason chosen. | √ |
| Selection of outcomes | 11 | Describe what outcomes were used as the measure(s) of benefit(s) and harm(s). | √ |
| Measurement of outcomes | 12 | Describe how outcomes used to capture benefit(s) and harm(s) were measured. | √ |
| Valuation of outcomes | 13 | Describe the population and methods used to measure and value outcomes. | √ |
| Measurement and valuation of resources  and costs | 14 | Describe how costs were valued. | √ |
| Currency, price date, and conversion | 15 | Report the dates of the estimated resource quantities and unit costs, plus the currency and year of conversion. | √ |
| Rationale and  description of model | 16 | If modelling is used, describe in detail and why used. Report if the model  is publicly available and where it can be accessed. | √ |
| Analytics and assumptions | 17 | Describe any methods for analysing or statistically transforming data, any extrapolation methods, and approaches for validating any model used. | √ |
| Characterizing heterogeneity | 18 | Describe any methods used for estimating how the results of the study vary for sub-groups. | √ |
| Characterizing  distributional effects | 19 | Describe how impacts are distributed across different individuals  or adjustments made to reflect priority populations. | √ |
| Characterizing uncertainty | 20 | Describe methods to characterize any sources of uncertainty in the analysis. | √ |
| Approach to engagement with patients and others affected by the study | 21 | Describe any approaches to engage patients or service recipients, the general public, communities, or stakeholders (e.g., clinicians or payers) in the design of the study. | √ |
| **RESULTS** | | |  |
| Study parameters | 22 | Report all analytic inputs (e.g., values, ranges, references) including uncertainty or distributional assumptions. | √ |
| Summary of main results | 23 | Report the mean values for the main categories of costs and outcomes of interest and summarise them in the most appropriate overall measure. | √ |
| Effect of uncertainty | 24 | Describe how uncertainty about analytic judgments, inputs, or projections  affect findings. Report the effect of choice of discount rate and time horizon, if applicable. | √ |
| Effect of engagement with patients and others affected by the study | 25 | Report on any difference patient/service recipient, general public, community, or stakeholder involvement made to the approach or findings of the study | √ |
| **DISCUSSION** | | |  |
| Study findings, limitations, generalizability, and current knowledge | 26 | Report key findings, limitations, ethical or equity considerations not captured, and how these could impact patients, policy, or practice. | √ |
|  | | | |
| Source of funding | 27 | Describe how the study was funded and any role of the funder in the identification, design, conduct, and reporting of the analysis | √ |
| Conflicts of interest | 28 | Report authors conflicts of interest according to journal or  International Committee of Medical Journal Editors requirements. | √ |

# **Table S2 Baseline characteristics of patients in DeLLphi-304**

|  | **Tarlatamab**  **(N = 254)** | **Chemotherapy**  **(N = 255)** |
| --- | --- | --- |
| **Characteristic** |  |  |
| **Sex — no. (%)** |  |  |
| **Male** | 182 (72) | 169 (66) |
| **Female** | 72 (28) | 86 (34) |
| **Race or ethnic group — no. (%)†** | | |
| **Asian** | 97 (38) | 107 (42) |
| **Black** | 2 (1) | 3 (1) |
| **White** |  |  |
| **Overall** | 152 (60) | 139 (55) |
| **Hispanic or Latino** | 12 (5) | 11 (4) |
| **Not Hispanic or Latino** | 140 (55) | 128 (50) |
| **Other‡** | 2 (1) | 4 (2) |
| **Missing** | 1 (<1) | 2 (1) |
| **Median age (range) — yr** | 64 (20–86) | 66 (26–84) |
| **ECOG performance-status score — no. (%)§** | | |
| **0** | 83 (33) | 80 (31) |
| **1** | 169 (67) | 173 (68) |
| **2** | 2 (1) | 2 (1) |
| **Smoking history — no. (%)** |  |  |
| **Never** | 23 (9) | 31 (12) |
| **Current** | 54 (21) | 51 (20) |
| **Former** | 177 (70) | 173 (68) |
| **Previous PD-L1 or PD-1 inhibitor therapy — no. (%)** | | |
| **Yes** | 180 (71) | 180 (71) |
| **No** | 74 (29) | 75 (29) |
| **Chemotherapy-free interval — no. (%)** | | |
| **<90 days** | 109 (43) | 114 (45) |
| **≥90 to <180 days** | 85 (33) | 78 (31) |
| **≥180 days** | 60 (24) | 63 (25) |
| **Brain metastases — no. (%)‖** | | |
| **Yes** | 113 (44) | 115 (45) |
| **No** | 141 (56) | 140 (55) |
| **Liver metastases — no. (%)** |  |  |
| **Yes** | 84 (33) | 95 (37) |
| **No** | 170 (67) | 160 (63) |
| **DLL3 expression — no./total no. (%)*** | 207/217 (95) | 198/214 (93) |

# **Table S3 Model choice for all survival data**

| **Population & Model** | **AIC** | **BIC** | **Δ AIC^#^** | **Weight^*^** | **Parameters** |
| --- | --- | --- | --- | --- | --- |
| **Tarlatamab-OS** |  |  |  |  |  |
| Gompertz | 898.601 | 905.676 | 0 | 35.06% | Shape=0.0395, Rate=0.0392 |
| Exponential | 899.402 | 902.94 | 0.801 | 23.49% | Rate=0.0495 |
| Weibull | 899.75 | 906.824 | 1.149 | 19.73% | Shape=1.1188, cale=18.8491 |
| Gamma | 899.966 | 907.041 | 1.365 | 17.71% | Shape=1.1430, Rate=0.0610 |
| Log-Logistic | 903.269 | 910.344 | 4.668 | 3.40% | Shape=1.2674, cale=14.2113 |
| Log-Normal | 906.771 | 913.846 | 8.17 | 0.59% | Meanlog=2.6960, Sdlog=1.4695 |
| Generalized Gamma | 1047.189 | 1059.374 | 148.588 | 0.00% | Mu=3.1701, Sigma=3.1701, Q=3.9652 |
| **Chemotherapy-OS** |  |  |  |  |  |
| Log-Logistic | 1048.894 | 1055.976 | 0 | 37.68% | Shape=1.4935, Scale=8.6937 |
| Log-Normal | 1049.021 | 1056.104 | 0.127 | 35.35% | Meanlog=2.1675, Sdlog=1.1802 |
| Generalized Gamma | 1050.444 | 1061.068 | 1.55 | 17.38% | Mu=2.2610, Sigma=1.1048, Q=0.2405 |
| Gamma | 1052.498 | 1059.581 | 3.604 | 6.22% | Shape=1.3318, Rate=0.1131 |
| Weibull | 1054.112 | 1061.195 | 5.218 | 2.78% | Shape=1.1980, Scale=12.3104 |
| Exponential | 1058.006 | 1061.547 | 9.112 | 0.39% | Rate=0.0767 |
| Gompertz | 1059.086 | 1066.168 | 10.192 | 0.23% | Shape=0.0211, Rate=0.0689 |
| **Tarlatamab-PFS** |  |  |  |  |  |
| Log-Logistic | 1089.338 | 1096.413 | 0 | 45.89% | Shape=1.4887, Scale=4.0541 |
| Log-Normal | 1089.644 | 1096.719 | 0.306 | 39.38% | Meanlog=1.4099, Sdlog=1.1597 |
| Generalized Gamma | 1091.602 | 1102.214 | 2.264 | 14.79% | Mu=1.3882, Sigma=1.1666, Q=-0.0442 |
| Gamma | 1109.25 | 1116.325 | 19.912 | 0.00% | Shape=1.1580, Rate=0.1836 |
| Gompertz | 1108.038 | 1115.112 | 18.7 | 0.00% | Shape=-0.0453, Rate=0.1823 |
| Exponential | 1109.818 | 1113.356 | 20.48 | 0.00% | Rate=0.1541 |
| Weibull | 1111.212 | 1118.286 | 21.874 | 0.00% | Shape=1.0478, Scale=6.4955 |
| **Chemotherapy-PFS** |  |  |  |  |  |
| Generalized Gamma | 1123.865 | 1134.489 | 0 | 50.00% | Mu=1.3666, Sigma=0.7909, Q=0.4193 |
| Gamma | 1124.605 | 1131.688 | 0.74 | 34.54% | Shape=1.8431, Rate=0.4166 |
| Log-Normal | 1127.017 | 1134.099 | 3.152 | 10.39% | Meanlog=1.2038, Sdlog=0.8422 |
| Log-Logistic | 1129.443 | 1136.525 | 5.578 | 3.08% | Shape=2.0491, Scale=3.4168 |
| Weibull | 1130.251 | 1137.334 | 6.386 | 2.06% | Shape=1.4161, Scale=4.8527 |
| Gompertz | 1149.62 | 1156.703 | 25.755 | 0.00% | Shape=0.1036, Rate=0.1538 |
| Exponential | 1166.168 | 1169.709 | 42.303 | 0.00% | Rate=0.2187 |

AIC,Akaike Information Criterion;BIC,Bayesian Information Criterion;OS,overall survival; PFS, progression-free survival.

**Δ AIC^#^:** Difference between the AIC of the specific model and the minimum AIC in that group.

**Weight^*^:** Calculated as$w_{i}=\frac{\exp\left( -0.5\times\Delta AIC_{i} \right)}{\sum\exp\left( -0.5\times\Delta AIC \right)}$.

The final survival probability S(t) used in the Markov trace was calculated as the weighted average of the survival probabilities from all candidate distributions at each cycle $S_{\mathrm{AICavg}}\left( t \right)=\sum_{i=1}^{n} w_{i}\cdot S_{i}\left( t \right)$

where $S_{i}\left( t \right)$ represents the survival probability of the $i$-th distribution at time $t$."

# **Table S4 Post-progression pathway inputs**

| **Country** | **Trial Arm** | **Status After Progression** | **Proportion of Cohort** | **Regimen Composition (Weight)** |
| --- | --- | --- | --- | --- |
| **United States** | **Tarlatamab** | Active Subsequent Therapy | 44% | Topotecan (77.5%) |
|  |  |  |  | Lurbinectedin (22.5%) |
|  |  |  |  | Pembrolizumab* (29.0%) |
|  |  | BSC | 56% | BSC (100%) |
|  | **Chemotherapy** | Active Subsequent Therapy | 49% | Lurbinectedin (77.5%) |
|  |  |  |  | Topotecan (22.5%) |
|  |  |  |  | Pembrolizumab* (29.0%) |
|  |  | BSC | 51% | BSC (100%) |
| **China** | **Tarlatamab** | Active Subsequent Therapy | 44% | Topotecan (100.0%) |
|  |  |  |  | Tislelizumab* (29.0%) |
|  |  | BSC | 56% | BSC (100%) |
|  | **Chemotherapy** | Active Subsequent Therapy | 49% | Anlotinib (71.0%) |
|  |  |  |  | Tislelizumab* (29.0%) |
|  |  | BSC | 51% | BSC (100%) |

Note: Proportions are derived from the DeLLphi-304 trial.

*A 29% utilization rate for PD1 was applied across all cohorts to reflect the exact proportion of enrolled patients who were naïve to prior programmed cell death protein 1 therapy.

Treatment costs accrue continuously during the progressed disease state until death.

# **Table S5 Basic parameters input to the model (US)**

| **Name** | Baseline value | Low | Upper | Distribution | **Price Year*** | Source |
| --- | --- | --- | --- | --- | --- | --- |
| **Costs*** |  |  |  |  |  |  |
| All iv therapies* | 144.09 | 115.272 | 172.908 | gamma | 2025 | (1) |
| Laboratory/time | 23.75 | 19 | 28.5 | gamma | 2023 | (2, 3) |
| abdomen CT | 359 | 287.2 | 430.8 | gamma | 2025 | (4) |
| Thorax CT | 182 | 145.6 | 218.4 | gamma | 2025 | (4) |
| Brain MRI | 337 | 269.6 | 404.4 | gamma | 2025 | (4) |
| Bone scan | 344 | 275.2 | 412.8 | gamma | 2025 | (4) |
| Cost of best supportive | 781.58 | 625.26 | 937.90 | gamma | 2019 | (3, 5) |
| End of life cost | 11,573.94 | 9,259.15 | 13,888.73 | gamma | 2019 | (3, 5) |
| Cost of FN/cycle | 35,089.25 | 28071.4 | 42,107.1 | gamma | 2022 | (3, 6) |
| Cost of Thrombocytopenia/cycle | 23,880.58 | 19,104.46 | 28,656.70 | gamma | 2022 | (3, 6) |
| Cost of Leukopenia/cycle | 23,355.17 | 18,684.14 | 28,026.20 | gamma | 2022 | (3, 6) |
| Cost of Neutropenia/cycle | 23,355.17 | 18,684.14 | 28,026.20 | gamma | 2022 | (3, 6) |
| Cost of anemia/cycle | 18,759.84 | 15,007.87 | 22,511.81 | gamma | 2022 | (3, 6) |
| Cost of CRS(Grade 1/2)/cycle | 16,063 | 2,221 | 29,473 | gamma | 2021 | (3, 7-9) |
| Cost of CRS(Grade 3/4)/cycle | 148,251 | 17,609 | 315,629 | gamma | 2021 | (3, 7-9) |
| **Utility** |  |  |  |  |  |  |
| Utility of PFS | 0.7 | 0.56 | 0.84 | beta | - | (10) |
| Utility of PD | 0.6 | 0.48 | 0.72 | beta | - | (10) |
| **Disutility of AE** |  |  |  |  |  |  |
| Disutility of FN | 0.33 | 0.26 | 0.40 | beta | - | (11) |
| Disutility of Thrombocytopenia | 0.05 | 0.04 | 0.06 | beta | - | (12) |
| Disutility of Leukopenia | 0.09 | 0.07 | 0.11 | beta | - | (13) |
| Disutility of Neutropenia | 0.09 | 0.07 | 0.11 | beta | - | (13) |
| Disutility of Anemia | 0.07 | 0.06 | 0.08 | beta | - | (13) |
| Disutility of CRS(Grade 1) | 0.01 | 0 | 0.12 | beta | - | (14) |
| Disutility of CRS(Grade 2) | 0.05 | 0.04 | 0.06 | beta | - | (14) |
| Disutility of CRS(Grade 3/4) | 0.23 | 0.18 | 0.28 | beta | - | (14) |
| **Risk of AE** |  |  |  |  |  |  |
| **Tarlatamab group** |  |  |  |  |  |  |
| Febrile neutropenia | 2% | 1.60% | 2.40% | beta | - | (15) |
| Thrombocytopenia | 1% | 0.80% | 1.20% | beta | - | (15) |
| Leukopenia | 2% | 1.60% | 2.40% | beta | - | (15) |
| Neutropenia | 6% | 4.80% | 7.20% | beta | - | (15) |
| Anemia | 4% | 3.20% | 4.80% | beta | - | (15) |
| CRS (Grade 1) | 42% | 33.6% | 50.4% | beta | - | (15) |
| CRS (Grade 2) | 13% | 10.4% | 15.6% | beta | - | (15) |
| CRS (Grade 3/4) | 1% | 0.8% | 1.2% | beta | - | (15) |
| **Chemotherapy group** |  |  |  |  |  |  |
| Febrile neutropenia | 11% | 8.80% | 13.20% | beta | - | (15) |
| Thrombocytopenia | 11% | 8.80% | 13.20% | beta | - | (15) |
| Leukopenia | 14% | 11.20% | 16.80% | beta | - | (15) |
| Neutropenia | 23% | 18.40% | 27.60% | beta | - | (15) |
| Anemia | 29% | 23.20% | 34.80% | beta | - | (15) |
| **Cost of drugs (per cycle)** |  |  |  |  |  |  |
| Tarlatamab(1mg) | 1567.49 | 1253.99 | 1880.99 | gamma | 2025 | (16) |
| Topotecan (0.1mg) | 1.342 | 1.07 | 1.61 | gamma | 2025 | (16) |
| Lurbinectedin(0.1mg) | 206.13 | 164.90 | 247.36 | gamma | 2025 | (16) |
| Pembrolizumab(1mg) | 58.56 | 46.85 | 70.27 | gamma | 2025 | (16) |
| **Other** |  |  |  |  |  |  |
| **Discount for cost and utility** | 0.03 | 0 | 0.08 | beta | - | (17) |
| **Weight(kg)** |  |  |  |  |  |  |
| Man | 90.63 | 72.50 | 108.76 | normal | - | (18) |
| Woman | 77.47 | 61.98 | 92.96 | normal | - | (18) |
| **Height(cm)** |  |  |  |  |  | (18) |
| Man | 175.26 | 140.21 | 210.31 | normal | - | (18) |
| Woman | 161.29 | 129.03 | 193.55 | normal | - | (18) |

IV, Intravenous Injection; CT, computed tomography; MRI ,magnetic resonance imaging; FN, febrile neutropenia; PFS, progression-free survival; PD, progressed disease; AE, adverse events.

Note: Price Year indicates the original data year. Historical U.S. costs were inflated to 2025 values using the Medical Care Component of the Consumer Price Index . Costs from 2025 were used directly without adjustment.

# **Table S6 Basic parameters input to the model (China)**

| **Name** | Baseline value | Low | Upper | Distribution | **Price Year*** | Source |
| --- | --- | --- | --- | --- | --- | --- |
| **Costs($)** |  |  |  |  |  |  |
| All iv therapies* | 144.09 | 115.27 | 172.91 | gamma | 2024 | (19-21) |
| Laboratory/time | 23.75 | 19.00 | 28.50 | gamma | 2024 | (19-21) |
| abdomen CT | 359 | 287.20 | 430.80 | gamma | 2024 | (19-21) |
| Thorax CT | 182 | 145.60 | 218.40 | gamma | 2024 | (19-21) |
| Brain MRI | 337 | 269.60 | 404.40 | gamma | 2024 | (19-21) |
| Bone scan | 344 | 275.20 | 412.80 | gamma | 2024 | (19-21) |
| Cost of best supportive | 781.58 | 625.26 | 937.90 | gamma | 2019 | (22, 23) |
| End of life cost | 11,573.94 | 9,259.15 | 13,888.73 | gamma | 2012 | (23, 24) |
| Cost of FN/cycle | 7158.10 | 5,726.48 | 8,589.72 | gamma | 2021 | (23, 25) |
| Cost of Thrombocytopenia/cycle | 1505.92 | 1,204.74 | 1,807.10 | gamma | 2022 | (23, 26) |
| Cost of Leukopenia/cycle | 115.01 | 92.01 | 138.01 | gamma | 2022 | (23, 26) |
| Cost of Neutropenia/cycle | 115.01 | 92.01 | 138.01 | gamma | 2022 | (23, 26) |
| Cost of anemia/cycle | 138.75 | 111.00 | 166.50 | gamma | 2022 | (23, 26) |
| Cost of CRS(Grade 1/2)/cycle | 10,157 | 2,954 | 104,733 | gamma | 2020 | (23, 27) |
| Cost of CRS(Grade 3/4)/cycle | 31,935 | 9,522 | 190,510 | gamma | 2020 | (23, 27) |
| **Utility** |  |  |  |  |  |  |
| Utility of PFS | 0.7 | 0.56 | 0.84 | beta | - | (10) |
| Utility of PD | 0.6 | 0.48 | 0.72 | beta | - | (10) |
| **Disutility of AE** |  |  |  |  |  |  |
| Disutility of FN | 0.33 | 0.26 | 0.40 | beta | - | (11) |
| Disutility of Thrombocytopenia | 0.05 | 0.04 | 0.06 | beta | - | (12) |
| Disutility of Leukopenia | 0.09 | 0.07 | 0.11 | beta | - | (13) |
| Disutility of Neutropenia | 0.09 | 0.07 | 0.11 | beta | - | (13) |
| Disutility of Anemia | 0.07 | 0.06 | 0.08 | beta | - | (13) |
| Disutility of CRS(Grade 1) | 0.01 | 0 | 0.12 | beta | - | (14) |
| Disutility of CRS(Grade 2) | 0.05 | 0.04 | 0.06 | beta | - | (14) |
| Disutility of CRS(Grade 3/4) | 0.23 | 0.18 | 0.28 | beta | - | (14) |
| **Risk of AE** |  |  |  |  |  |  |
| **Tarlatamab group** |  |  |  |  |  |  |
| Febrile neutropenia | 2% | 1.60% | 2.40% | beta | - | (15) |
| Thrombocytopenia | 1% | 0.80% | 1.20% | beta | - | (15) |
| Leukopenia | 2% | 1.60% | 2.40% | beta | - | (15) |
| Neutropenia | 6% | 4.80% | 7.20% | beta | - | (15) |
| Anemia | 4% | 3.20% | 4.80% | beta | - | (15) |
| CRS (Grade 1) | 42% | 33.6% | 50.4% | beta | - | (15) |
| CRS (Grade 2) | 13% | 10.4% | 15.6% | beta | - | (15) |
| CRS (Grade 3/4) | 1% | 0.8% | 1.2% | beta | - | (15) |
| **Chemotherapy group** |  |  |  |  |  |  |
| Febrile neutropenia | 11% | 8.80% | 13.20% | beta | - | (15) |
| Thrombocytopenia | 11% | 8.80% | 13.20% | beta | - | (15) |
| Leukopenia | 14% | 11.20% | 16.80% | beta | - | (15) |
| Neutropenia | 23% | 18.40% | 27.60% | beta | - | (15) |
| Anemia | 29% | 23.20% | 34.80% | beta | - | (15) |
| **Cost of drugs (per cycle,$)** |  |  |  |  |  |  |
| Tarlatamab(1mg) | 522.50 | 418.00 | 627.70 | gamma | 2025 | (16, 28) |
| Topotecan (0.1mg) | 7.19 | 5.75 | 8.63 | gamma | 2025 | (29) |
| Pembrolizumab(1mg) | 25.11 | 20.09 | 30.13 | gamma | 2025 | (29) |
| Tislelizumab | 351.27 | 281.02 | 421.52 | gamma | 2025 | (29) |
| Anlotinib (12mg) | 277.81 | 222.25 | 333.37 | gamma | 2025 | (29) |
| **Other** |  |  |  |  |  |  |
| **Discount for cost and utility** | 0.05 | 0 | 0.08 | beta | - | (30) |
| **Weight(kg)** |  |  |  |  |  |  |
| Man | 69.0 | 55.20 | 82.80 | normal | - | (31) |
| Woman | 60.3 | 48.24 | 72.36 | normal | - | (31) |
| **Height(cm)** |  |  |  |  |  |  |
| Man | 165.9 | 132.72 | 199.08 | normal | - | (31) |
| Woman | 155.1 | 124.08 | 186.12 | normal | - | (31) |

IV, Intravenous Injection; CT, computed tomography; MRI, magnetic resonance imaging; FN, Febrile Neutropenia; PFS, progression-free survival; PD, progressed disease; AE, adverse events.

Note: Chinese costs were inflated to 2025 values using the medical-specific Consumer Price Index and subsequently converted to U.S. dollars at an exchange rate of RMB 7.1371 per dollar.

# **Table S7 Subgroups Analyses**

| **Subgroups** | **HR for OS**  **(95% CI)** | **HR for PFS**  **(95% CI)** | **ICER1^a^**  **$/QALY (range)** | **CE**  **Probability,%** | **ICER2^a^**  **$/QALY (range)** | **CE**  **Probability,%** |
| --- | --- | --- | --- | --- | --- | --- |
| **All patients** | 0.60  (0.47,0.77) | 0.71  (0.59,0.86) | 150,000.93  (83,503.32-240,939.98) | 52% | 40,247.35  (20,615.35-62,728.95) | 50.4% |
| **Age, years** |  |  |  |  |  |  |
| <65 | 0.57  (0.40,0.81) | 0.77  (0.59,1.01) | 134,084.66  (68,097.26-265,381.41) | 94.6% | 36,543.27  (16,462.98-69,260.98) | 89.5% |
| ≥65 | 0.67  (0.48,0.94) | 0.65  (0.50,0.86) | 152,263.20  (106,392.05-405,721.49) | 43.7% | 39,958.65  (29,400.06-102,468.51) | 55.1% |
| **Sex** |  |  |  |  |  |  |
| Male | 0.70  (0.53,0.93) | 0.77  (0.62,0.97) | 170,163.05  (104,034.58 -367,821.78) | 2.2% | 46,972.82  (29,703.73-97,899.06) | 0.6% |
| Female | 0.43  (0.26,0.72) | 0.61  (0.42,0.87) | 129,061.82  (79,230.52-258,232.80) | 96.7% | 33,067.35  (21,319.10-62,330.97) | 99.2% |
| **Region** |  |  |  |  |  |  |
| White | 0.51  (0.37,0.70) | 0.79  (0.61,1.02) | 119,337.32  (79,827.86-202,347.46) | 99.8% | 32,502.98  (22,456.79-52,850.55) | 99.8% |
| Asian | 0.75  (0.50,1.11) | 0.69  (0.51,0.94) | 206,221.44  (103,911.91-806,325.90) | 0% | 55,662.99  (29,518.82-206,122.96) | 0% |
| **Prior anti-PD-(L)1 exposure** | | | |  |  |  |
| Yes | 0.61  (0.45,0.82) | 0.67  (0.53,0.84) | 159,420.76  (103,784.92 -288,948.68) | 19.3% | 42,253.36  (28,433.14-73,580.29) | 25.7% |
| No | 0.65  (0.42,1.03) | 0.89  (0.62,1.28) | 137,850.13  (71,308.04-dominated) | 87.6% | 39,224.83  (21,130.35-dominated) | 65.7% |
| **Chemotherapy-free interval** | | | |  |  |  |
| <90 days | 0.60  (0.43,0.84) | 0.71  (0.54,0.94) | 150,000.93  (92,140.03-300,950.32) | 52% | 40,247.35  (25,753.72-77,044.13) | 50.1% |
| ≥90 days | 0.65  (0.45,0.93) | 0.73  (0.56,0.95) | 160,708.19  (93,930.51-381,615.41) | 14.1% | 43,592.56  (26,386.94- 98,986.40) | 11.3% |
| ≥90 to <180 days | 0.71  (0.46,1.10) | 0.74  (0.52,1.04) | 179,168.79  (88,520.30-774,622.75) | 0.1% | 49,025.91  (25,367.99- 198,839.54) | 0.1% |
| ≥180 days | 0.54  (0.29,1.03) | 0.74  (0.49,1.12) | 131,699.49  (67,714.49-55,4091.68) | 96.1% | 35,471.19  (19,097.61- 139,817.08) | 95.2% |
| **Brain metastases** | | | |  |  |  |
| Yes | 0.45  (0.31,0.65) | 0.60  (0.45,0.80) | 134,260.42  (89,649.79 -221,235.03) | 92.1% | 34,335.62  (23,920.06-53,991.31) | 97.9% |
| No | 0.81  (0.58,1.13) | 0.83  (0.64,1.08) | 207,216.86  (103,184.20-1,078,827.86) | 0% | 58,970.46  (30,425.07- 293,013.03) | 0% |
| **Liver metastases** | | | |  |  |  |
| Yes | 0.82  (0.57,1.18) | 0.84  (0.62,1.14) | 210,845.29  (96,981.20-1,880,661.45) | 0% | 60,262.14  (28,902.50- 507,531.25) | 0% |
| No | 0.54  (0.39,0.75) | 0.69  (0.54,0.88) | 138,792.37  (92,055.78-243,333.77) | 87.7% | 36,811.10  (25,258.68- 62,007.27) | 91% |
| **Chemotherapy** | | | |  |  |  |
| Topotecan/Amrubicin | 0.57  (0.44,0.75) | 0.76  (0.62,0.94) | 135,427.14  (93,422.42-222,686.07) | 91.8% | 36,799.79  (26,150.04-58,581.16) | 90% |
| Lurbinectedin | 0.81  (0.46,1.44) | 0.56  (0.34,0.90) | 273,824.40  (99,560.19-273,824.40) | 0% | 70,588.60  (27,700.40-70,588.60) | 0% |

Costs are in US dollars

CI, confidence interval; HR, hazard ratio; ICER, incremental cost-effectiveness ratio; OS, overall survival;PD-L1, programmed cell death ligand 1; QALY, quality-adjusted life year; US ,United States, CE, cost effectiveness

^a^ICER1 is calculated based in the US; price of Tarlatamab= $704.99/1 mg; WTP = $150,000

^b^ICER2 is calculated based in the China; price of Tarlatamab= $ 196.55/1mg; WTP = $40247.01

^c^“Dominated” reveals that a plan is an absolute disadvantaged one

# **Table S8 Scenario Analysis**

| **Scenario** | **US** | | **CN** | |
| --- | --- | --- | --- | --- |
|  | **ICER($)** | **PSA 95% UI** | **ICER($)** | **PSA 95% UI** |
| **Model** |  |  |  |  |
| Exponential | 382258.61 | 384,034.85 (333,924.05 -445,907.18) | 120,791.03 | 121,198.93(105,935.08-139,634.05) |
| Gama | 393407.24 | 395,554.72(344,226.68-452,406.77) | 12,6,063.43 | 126,825.00(111,043.70 -147,678.87) |
| Gompertz | 712383.64 | 719,083.06(625,164.59 -867,461.14) | 222,079.86 | 222,422.7(193,373.33-264,392.46) |
| Weibull | 417736.22 | 420,954.19(367,571.69-483,614.78) | 133,776.164 | 134,492.46 (117,902.27-154,615.86) |
| Log-logistic | 214944.20 | 216,460.38 (189,298.46-251,034.94) | 64,993.28 | 65,322.78(57,316.98-76,489.17) |
| Log-normal | 172409.35 | 173,362.35(149,322.24-205,865.58) | 52,136.61 | 52,626.08(45,457.42-61,719.43) |
| Gen-gamma | Dominated | 8,082,345.81(-93,714,453.65-90,983,880.36) | Dominated | Dominated(Dominated- 23,414,574.67) |
| **AEs** | 1293252.19 | 1,352,874.27(944,044.27-2,094,511.66) | 402,306.59 | 413,180.33(293,472.27-630,297.64) |
| **CRS** | 1335355.43 | 1,405,710.86(966,333.42-2,242,121.88) | 435,227.27 | 459,454.86(318,535.06-727,269.94) |
| **Mortality Ratio^*^** | 617,696.81 | 623,093.36(532,402.21-754,570.04) | 194,264.33 | 196,232.75(168,603.38-236,985.44) |
| **5 years’ time** | 912,977.97 | 928,053.14(746,393.39-1,218,046.75) | 279,920.86 | 283,913.78(230,426.65-378,865.52) |
| **10 years’ time** | 1,113,951.71 | 1,148,886.72(857,029.18-1,639,953.11) | 340,219.23 | 346,613.46(266,243.07-491,801.95) |

**^*^**This scenario analysis, we utilized a Standardized Mortality Ratio (SMR) of 1.5 to scale the background mortality rate within the PFS state.

# **References**

1. "CMS.gov.Physician Fee Schedule." <https://www.cms.gov/medicare/physician-fee-schedule/search?Y=3&T=0&HT=0&CT=3&H1=80076&M=5>, accessed 15 August, 2025.

2. Shao T, Zhao M, Liang L, Tang W: Serplulimab Plus Chemotherapy vs Chemotherapy for Treatment of US and Chinese Patients with Extensive-Stage Small-Cell Lung Cancer: A Cost-Effectiveness Analysis to Inform Drug Pricing. BioDrugs 37:421-32, 2023. <https://doi.org/10.1007/s40259-023-00586-6>.

3. "Consumer price index. United States Bureau of Labor Statistics." <https://www.bls.gov/cpi/>. accessed 15 August, 2025.

4. "Medicare.gov." <https://www.medicare.gov/procedure-price-lookup/>, accessed 15 August, 2025.

5. Criss SD, Mooradian MJ, Watson TR, et al.: Cost-effectiveness of Atezolizumab Combination Therapy for First-Line Treatment of Metastatic Nonsquamous Non-Small Cell Lung Cancer in the United States. JAMA Netw Open 2:e1911952, 2019. <https://doi.org/10.1001/jamanetworkopen.2019.11952>.

6. "The Healthcare Cost and Utilization Project (HCUP) ", <https://hcupnet.ahrq.gov/#setup>, accessed 15 August, 2025.

7. Abramson JS, Siddiqi T, Garcia J, et al.: Cytokine release syndrome and neurological event costs in lisocabtagene maraleucel-treated patients in the TRANSCEND NHL 001 trial. Blood Adv 5:1695-705, 2021. <https://doi.org/10.1182/bloodadvances.2020003531>.

8. Saunders AC, Badaracco J: An economic model to estimate costs of cytokine release syndrome and neurological events among patients treated with CAR T cell therapies for relapsed or refractory follicular lymphoma. Blood 142:7247, 2023.

9. Logan AC, Shah BD, Pantin J, et al.: An Economic Model Comparing the Costs Associated with Cytokine Release Syndrome (CRS) and Immune Effector Cell-Associated Neurotoxicity Syndrome (ICANS) Among Patients Treated with Chimeric Antigen Receptor (CAR) T-Cell Therapies for Relapsed/Refractory B-Cell Acute Lymphoblastic Leukemia (R/R B-ALL). Transplantation and Cellular Therapy 31:S226-S27, 2025.

10. Vedadi A, Shakik S, Brown MC, et al.: The impact of symptoms and comorbidity on health utility scores and health-related quality of life in small cell lung cancer using real world data. Qual Life Res 30:445-54, 2021. <https://doi.org/10.1007/s11136-020-02615-1>.

11. Lee EK, Wong WW, Trudeau ME, Chan KK: Cost-effectiveness of prophylactic granulocyte colony-stimulating factor for febrile neutropenia in breast cancer patients receiving FEC-D. Breast Cancer Res Treat 150:169-80, 2015. <https://doi.org/10.1007/s10549-015-3309-3>.

12. Nafees B, Lloyd AJ, Dewilde S, et al.: Health state utilities in non-small cell lung cancer: An international study. Asia Pac J Clin Oncol 13:e195-e203, 2017. <https://doi.org/10.1111/ajco.12477>.

13. Yang SC, Kuo CW, Lai WW, et al.: Dynamic Changes of Health Utility in Lung Cancer Patients Receiving Different Treatments: A 7-Year Follow-up. J Thorac Oncol 14:1892-900, 2019. <https://doi.org/10.1016/j.jtho.2019.07.007>.

14. Howell TA, Matza LS, Jun MP, et al.: Health State Utilities for Adverse Events Associated with Chimeric Antigen Receptor T-Cell Therapy in Large B-Cell Lymphoma. Pharmacoecon Open 6:367-76, 2022. <https://doi.org/10.1007/s41669-021-00316-0>.

15. Mountzios G, Sun L, Cho BC, et al.: Tarlatamab in Small-Cell Lung Cancer after Platinum-Based Chemotherapy. N Engl J Med 393:349-61, 2025. <https://doi.org/10.1056/NEJMoa2502099>.

16. "CMS.gov.Medicare Part B Drug Average Sales Price." <https://www.cms.gov/medicare/payment/fee-for-service-providers/part-b-drugs/average-drug-sales-price>, accessed 15 August, 2025.

17. Neumann PJ, Cohen JT, Weinstein MC: Updating cost-effectiveness--the curious resilience of the $50,000-per-QALY threshold. N Engl J Med 371:796-7, 2014. <https://doi.org/10.1056/NEJMp1405158>.

18. "Body Measurements.", <https://www.cdc.gov/nchs/fastats/body-measurements.htm>, accessed May 20, 2025.

19. "National Healthcare Security Administration." <https://www.nhsa.gov.cn/col/col202/index.html>, accessed December 15, 2025.

20. "Hunan Healthcare Security Administration." <https://ybj.hunan.gov.cn/ybj/first113541/firstF/f2113606/202404/t20240430_33291173.html>, accessed December 15, 2025.

21. "Zhejiang Provincial Medical Security Bureau." <http://ybj.zj.gov.cn/art/2025/10/20/art_1229225636_5663046.html>, accessed December 15, 2025.

22. Gu X, Zhang Q, Chu Y, et al.: Cost-effectiveness of afatinib, gefitinib, erlotinib and pemetrexed-based chemotherapy as first-line treatments for advanced non-small cell lung cancer in China. 127:84-89, 2019. <https://doi.org/10.1016/j.lungcan.2018.11.029>.

23. "National Bureau of Statistice.National data." <https://data.stats.gov.cn/english/easyquery.htm?cn=C01>, accessed 15 August, 2025.

24. Zeng X, Karnon J, Wang S, et al.: The cost of treating advanced non-small cell lung cancer: estimates from the chinese experience. PLoS One 7:e48323, 2012. <https://doi.org/10.1371/journal.pone.0048323>.

25. Wu Q, Li Q, Zhang J, et al.: Comparison of Primary and Secondary Prophylaxis Using PEGylated Recombinant Human Granulocyte–Stimulating Factor as a Cost-Effective Measure in Malignant Neoplasms: A Multicenter Retrospective Study. 12, 2021. <https://doi.org/10.3389/fphar.2021.690874>.

26. Rui M, Fei Z, Wang Y, et al.: Cost-effectiveness analysis of sintilimab + chemotherapy versus camrelizumab + chemotherapy for the treatment of first-line locally advanced or metastatic nonsquamous NSCLC in China. J Med Econ 25:618-29, 2022. <https://doi.org/10.1080/13696998.2022.2071066>.

27. Zhu F, Wei G, Zhang M, et al.: Factors Associated with Costs in Chimeric Antigen Receptor T-Cell Therapy for Patients with Relapsed/Refractory B-Cell Malignancies. Cell Transplant 29:963689720919434, 2020. <https://doi.org/10.1177/0963689720919434>.

28. "Assistant Secretary for Planning and Evaluation.Comparing Prescription Drugs in the U.S. and Other Countries: Prices and Availability.".<https://aspe.hhs.gov/reports/comparing-prescription-drugs>, accessed August 15, 2025.

29. "DrugDataexpy." <https://data.yaozh.com/>. accessed 15 August, 2025.

30. Guoen L. Chinese Guidelines for Pharmacoeconomics Evaluation 2020. China Market Press; 2020.

31. "General Administration of Sport of China. Fifth National physical fitness monitoring bulletin (2022)." <https://www.sport.gov.cn/n315/n329/c24335066/content.html>, accessed August 15, 2025.
